# GATA1-deficient human pluripotent stem cells generate neutrophils with improved antifungal immunity that is mediated by the integrin CD18

**DOI:** 10.1371/journal.ppat.1012654

**Published:** 2025-02-03

**Authors:** Andrew S. Wagner, Frances M. Smith, David A. Bennin, James A. Votava, Rupsa Datta, Morgan A. Giese, Wenxuan Zhao, Melissa C. Skala, Jing Fan, Nancy P. Keller, Anna Huttenlocher

**Affiliations:** 1 Department of Medical Microbiology and Immunology, University of Wisconsin School of Medicine and Public Health, Madison, Wisconsin, United States of America; 2 Department of Pediatrics, University of Wisconsin-Madison School of Medicine and Public Health, Madison, Wisconsin, United States of America; 3 Morgridge Institute for Research, Madison, Wisconsin, United States of America; 4 Department of Plant Pathology, University of Wisconsin-Madison, Madison, Wisconsin, United States of America; University of California Irvine School of Medicine, UNITED STATES OF AMERICA

## Abstract

Neutrophils are critical for host defense against fungi. However, the short life span and lack of genetic tractability of primary human neutrophils has limited *in vitro* analysis of neutrophil-fungal interactions. Human induced pluripotent stem cell (iPSC)-derived neutrophils (iNeutrophils) provide a genetically tractable system to study host defense responses of human neutrophils. Here, we show that deletion of the transcription factor GATA1 from human iPSCs results in iNeutrophils with improved antifungal activity against *Aspergillus fumigatus*. GATA1-knockout (KO) iNeutrophils have increased maturation, antifungal pattern recognition receptor expression and have improved neutrophil effector functions compared to wild-type iNeutrophils. iNeutrophils also show a shift in their metabolism following stimulation with fungal β-glucan to the pentose phosphate pathway (PPP), similar to primary human neutrophils. Furthermore, we show that deletion of the integrin CD18 attenuates the ability of GATA1-KO iNeutrophils to kill *A. fumigatus* but is not necessary for the metabolic shift*.* Collectively, these findings support iNeutrophils as a robust system to study human neutrophil antifungal immunity and has identified specific roles for CD18 in the defense response*.*

## Author summary

Neutrophils are important first responders to fungal infections, and understanding their antifungal functions is essential to better understanding of host defense responses. Primary human neutrophils are short lived and do not permit genetic manipulation, limiting their use to study neutrophil-fungal interactions *in vitro*. Human induced pluripotent stem cell (iPSC)-derived neutrophils (iNeutrophils) are a genetically tractable alternative to primary human neutrophils for *in vitro* analyses. In this report we show that GATA1-deficient iPSCs generate neutrophils (iNeutrophils) that are more mature than wild-type iNeutrophils and display increased antifungal activity against the human fungal pathogen *Aspergillus fumigatus*. We also show that GATA1-deficient iNeutrophils have increased expression of antifungal receptors than wild-type cells and shift their metabolism and execute neutrophil antifungal functions at levels comparable to primary human neutrophils. Deletion of the integrin CD18 blocks the ability of GATA1-deficient iNeutrophils to kill and control the growth of *A. fumigatus*, demonstrating an important role for this integrin in iNeutrophil antifungal activity. Collectively, these findings support the use of iNeutrophils as a model to study neutrophil antifungal immunity.

## Introduction

Invasive fungal infections (IFIs) are a significant health problem with greater than 1.5 million annual deaths worldwide [1]. Invasive aspergillosis (IA), primarily caused by *Aspergillus fumigatus,* is amongst the most common IFIs that requires hospitalization, and accounts for an estimated economic burden of 1.8 billion dollars within the United States alone [2]. Patients with neutropenia have increased risk of developing IA, since neutrophils act as important first responders at early stages of disease development [3]. Consequently, understanding how neutrophils interact with *A. fumigatus* is essential to elucidate the mechanisms of disease progression.

Neutrophils are key responders to *A. fumigatus* and express a repertoire of antifungal pattern recognition receptors (PRRs) that facilitate their interactions with invading fungi [4,5]. Once engaged, PRR activation stimulates neutrophils to perform numerous effector functions. These include antimicrobial activities like phagocytosis, reactive oxygen species (ROS) production, neutrophil extracellular trap (NET) extrusion, degranulation and the release of antimicrobial peptides such as elastase and lactoferrin to kill or attenuate fungal growth [6–10]. As part of their effector functions neutrophils rapidly produce ROS to mediate pathogen control [11–13], and recent evidence has demonstrated that metabolic reprogramming following activation towards the pentose phosphate pathway is necessary for the generation of more ROS during an oxidative burst [14,15]. Inhibiting the pentose pathway has also been shown to attenuate the antifungal activity against *A. fumigatus*, thus highlighting the importance of metabolic rewiring to control fungal growth [14]. Yet, our understanding of how human neutrophils actively sense fungal pathogens to direct metabolic changes, and how this in turn mediates their fungal killing capacity is incomplete. Primary neutrophils are short lived and are not genetically tractable [16], limiting the study of human neutrophil metabolism and host defense. Therefore, improved *in vitro* systems are needed to more effectively study fungal-neutrophil interaction dynamics.

Primary neutrophils from murine models harboring mutations of interest have been used to understand fungal defense responses. However, differences in murine granule composition, antimicrobial peptide production and overall antifungal activity have been reported [17]. Additionally, species-specific differences in the mechanisms used by neutrophils to mediate antifungal activity has complicated the translation of these findings to human neutrophils. For example, the ß(1,3)-glucan receptor dectin-1 is important in murine neutrophil antifungal activity [18,19], but reports suggest it is dispensable for human neutrophils [10,20]. These discrepancies highlight the need for genetically tractable human cell lines. Human myeloid cell lines such as HL-60 and PLB-985 cells undergo neutrophil-like differentiation *in vitro* and have also been used to study neutrophil antifungal immunity. Indeed, differentiated PLB-985 cells have recently been shown to phagocytose *A. fumigatus* conidia *in vitro* [21]. However, although PLB-985 and HL-60 cells can perform antimicrobial effector functions, their capacity to do so is attenuated, with reduced phagocytosis efficiency and NET release compared to primary human neutrophils [21,22]. Human induced pluripotent stem cell (iPSC)-derived neutrophils (iNeutrophils) are a newer source of genetically tractable neutrophils that can serve as an alternative to primary human neutrophils for functional assays. Indeed, these cells have already proven effective for studying mechanisms of neutrophil migration *in vitro* [23,24]. However, their application as a model cell line to study antimicrobial activity has been hampered by impaired functional capacity, at least in part due to their heterogeneity following maturation to iNeutrophils [25–27]. In light of this, it was recently discovered that deletion of the transcription factor GATA1 in iPSCs, which drives basophil and eosinophil differentiation during granulopoiesis, results in a more homogenous culture of mature iNeutrophils with multilobulated nuclei that display increased formation of NETs [28].

Here, we show that GATA1-knockout (KO) iNeutrophils have increased expression of antifungal PRRs and improved antifungal activity against *A. fumigatus* compared to wild-type (WT) cells*.* Like primary human neutrophils, iNeutrophils shift their metabolism towards the pentose phosphate pathway following stimulation with ß(1,3)-glucan rich bioparticles and GATA1-KO iNeutrophils show a reduced redox state by single cell metabolic imaging compared to WT cells. To identify mechanisms that regulate this metabolic shift and antifungal activity, we deleted the integrin receptor CD18, an integral subunit of the ß(1,3)-glucan binding protein complement receptor 3 (CR3). CD18-deficient GATA1-KO iNeutrophils had impaired ability to kill and control the growth of *A. fumigatus*. Although CD18 was not necessary for the shift in metabolism induced by ß-glucan, CD18 was necessary for their migratory response to fungi. Collectively, this work highlights the power of iNeutrophils to dissect mechanisms that regulate human neutrophil host defense and metabolism.

## Results

### GATA1-KO iNeutrophils have increased antifungal activity against *Aspergillus fumigatus*

**Fig 1:**
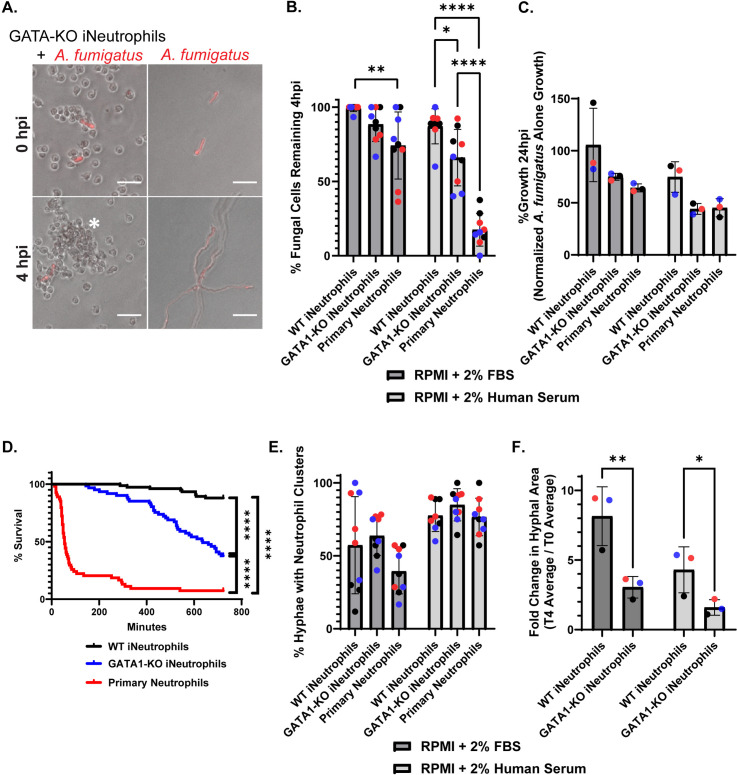
GATA1-KO iNeutrophils effectively kill and attenuate the growth of *A. fumigatus.* (A) Representative images of *A. fumigatus* growth in RPMI + 2% human serum in the presence and absence GATA1-KO iNeutrophils following 4 hours of incubation. (White asterisks denotes fungal killing as determined by loss of cytosolic *A. fumigatus* RFP signal)(Scale bar = 50μm). (B) Quantification of *A. fumigatus* survival at 4 hpi with either iNeutrophils or primary human neutrophils in RPMI media supplemented with 2% FBS or 2% human serum. Three biological replicates (indicated by different colors) with three technical replicates were assessed for all conditions tested. (n = 130 fungal cells analyzed for WT + 2% FBS, n = 127 fungal cells analyzed for WT + 2% human serum, n = 120 fungal cells analyzed for GATA1-KO + 2% FBS, n = 94 fungal cells analyzed for GATA1-KO + 2% human serum, n = 81 fungal cells analyzed for primary neutrophils + 2% FBS, n = 101 fungal cells analyzed for primary neutrophils + 2% human serum)(*p<0.05, **p<0.005, ****p<0.0001, via a two-way ANOVA with Sidak’s multiple comparison test) (C) Fungal growth determined via PrestoBlue viability staining following 24 hours of coincubation with either iNeutrophils or primary human neutrophils in RPMI media supplemented with 2% FBS or 2% human serum. Growth is normalized to *A. fumigatus* samples that were not treated with (i) Neutrophils in each condition. (n = 3 biological replicates)(Statistics determined via a two-way ANOVA with Sidak’s multiple comparison test). (D) Kaplan Meier survival curve of *A. fumigatus* following 12 hours of incubation with either iNeutrophils or primary human neutrophils in RPMI media supplemented with 2% human serum. Three biological replicates were run for all experimental conditions. (n = 75 fungal cells tracked for WT iNeutrophils, n = 61 fungal cells tracked for GATA1-KO iNeutrophils and n = 57 fungal cells tracked for primary human neutrophils)(****p<0.0001 via Cox proportional hazard regression analysis). (E) Quantification of *A. fumigatus* germlings displaying clusters of five or more (i)neutrophils aggregated around them at 4 hpi in RPMI media supplemented with 2% FBS or 2% human serum. Three biological replicates (indicated by different colors) with three technical replicates were assessed for all conditions tested. (n = 130 fungal cells analyzed for WT + 2% FBS, n = 127 fungal cells analyzed for WT + 2% human serum, n = 120 fungal cells analyzed for GATA1-KO + 2% FBS, n = 94 fungal cells analyzed for GATA1-KO + 2% human serum, n = 81 fungal cells analyzed for primary neutrophils + 2% FBS, n = 101 fungal cells analyzed for primary neutrophils + 2% human serum)(*p<0.05, via a two-way ANOVA with Sidak’s multiple comparison test). (F) Quantification of the fold change in hyphal growth of living fungal cells at 4 hpi relative to 0 hpi following incubation with either iNeutrophils or primary human neutrophils in RPMI media supplemented with 2% FBS or 2% human serum. (The average changes in fungal growth from 3 biological replicates is shown)(n = 114 fungal cells analyzed for WT + 2% FBS, n = 109 fungal cells analyzed for WT + 2% human serum, n = 108 fungal cells analyzed for GATA1-KO + 2% FBS, n = 64 fungal cells analyzed for GATA1-KO + 2% human serum)(*p<0.05, **p<0.005, via a two-way ANOVA with Sidak’s multiple comparison test).

Previous studies have demonstrated increased maturity of GATA1-KO iNeutrophils [28]. We therefore hypothesized that GATA1-KO cells may have increased antifungal activity *in vitro*. To test this, we generated a GATA1-KO iPSC line (S1 Fig) and differentiated these cells to iNeutrophils to compare the antifungal activity of WT and KO iNeutrophils against the common human fungal pathogen *A. fumigatus* strain CEA10. This fungal isolate expresses a cytoplasmic red fluorescence protein (RFP), which was used to track fungal viability (as indicated by the presence/absence of RFP signal) and growth following co-incubation with either iNeutrophils or primary human neutrophils (Fig 1A) [29]. Opsonization by both human iC3b and IgG antibodies have also been shown to impact neutrophil antifungal immune responses [10,30]. Therefore, to assess the impact that opsonization has on antifungal activity, assays were done in media supplemented with either 2% fetal bovine serum (FBS), which is low in human opsonins, or 2% human serum, which is high in human opsonins. By 4 hours post-incubation (hpi), GATA1-KO iNeutrophils showed increased fungal clearance in both FBS and human serum treated conditions (Fig 1B). This difference in fungal killing was most apparent in human serum supplemented media, where the mean fungal killing at 4hpi of WT iNeutrophils was ~13% as opposed to ~40% of fungal cells killed in GATA1-KO iNeutrophil treated conditions. Yet, it is important to note that by 4hpi, GATA1-KO iNeutrophils were still significantly less effective at clearing *A. fumigatus* germlings than primary human neutrophils. Fungal growth at 24hpi revealed that GATA1-KO iNeutrophils attenuated *A. fumigatus* hyphal development at levels comparable to primary human neutrophils (Fig 1C), although at this time point statistically significant differences were not observed between any of the cell lines tested. These findings suggest that the rate at which fungal killing occurs is likely slower for GATA1-KO iNeutrophils compared to primary neutrophils. Indeed, time lapse microscopy revealed a significant delay in fungal killing of GATA1-KO iNeutrophils compared to primary human cells over a 12-hour incubation period (Fig 1D and S1 Movie). Nonetheless, GATA1-KO iNeutrophils still killed ~70% of all fungal cells tracked, thus demonstrating potent antifungal activity *in vitro*.

The ability of neutrophils to actively sense and aggregate around developing hyphae is essential to their antifungal activity [31,32]. To provide insight into why GATA1-KO iNeutrophils more effectively kill *A. fumigatus* than WT cells, we assessed the ability of this cell line to cluster around developing hyphae following 4 hours of incubation. Overall, the percentage of fungal cells that displayed associated neutrophil clusters was increased in human serum supplemented conditions compared to FBS supplemented media, but no significant differences were observed when analyzing the number of clusters between WT and GATA1-KO iNeutrophils (Fig 1E). However, the fungal cells that remained at 4hpi were significantly smaller in GATA1-KO iNeutrophil treated samples compared to WT samples (Fig 1F). Thus, although both iNeutrophil lines aggregate around developing *A. fumigatus* hyphae at similar rates, their ability to control fungal growth within these clusters is improved with GATA1-KO iNeutrophils.

### GATA1-KO iNeutrophils display increased cell surface expression of antifungal PRRs

**Fig 2:**
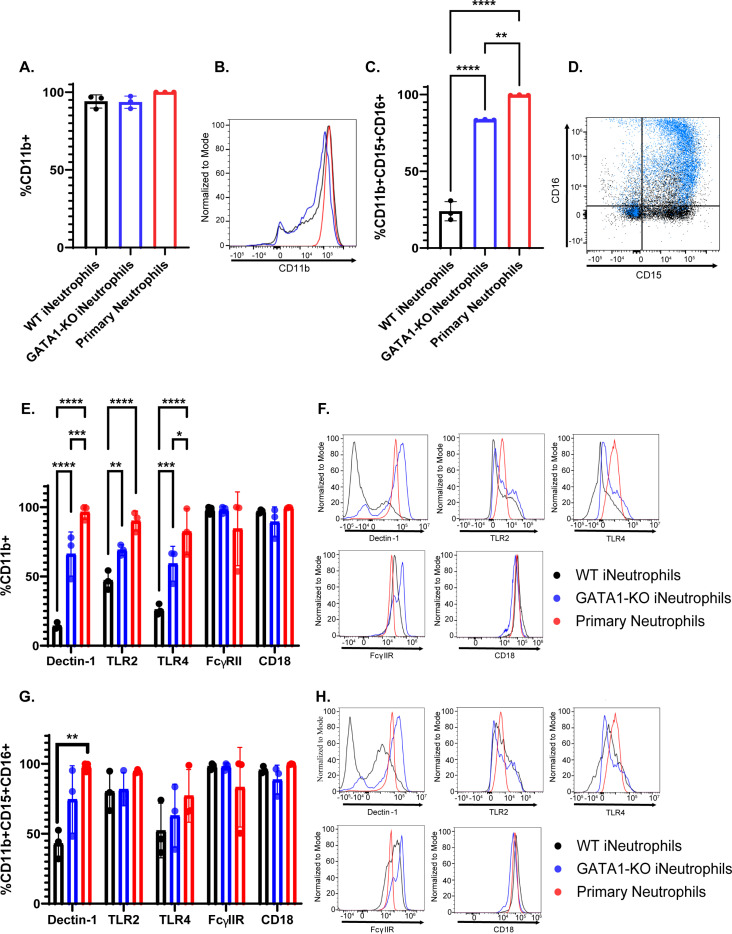
GATA1-KO iNeutrophils display increased surface marker expression of antifungal pattern recognition receptors. (A) Quantification of the percentage of cells expressing CD11b in both iNeutrophils and primary human neutrophils (n = 3 biological replicates)(Statistics run via one-way ANOVA with Tukey’s multiple comparison analysis). (B) Representative histogram of CD11b expression intensity on all cell lines stained. Colors of each sample match those shown in 2A. (C) Quantification of the percentage of cells expressing CD11b, CD15 and CD16 in both iNeutrophils and primary human neutrophils (n = 3 biological replicates)(**p<0.005, ****p<0.0001, via one-way ANOVA with Tukey’s multiple comparison analysis). (D) Representative scatter plot of CD15 and CD16 expression profiles of WT (black) and GATA1-KO iNeutrophils (blue). (E) Quantification of the percentage of CD11b+ cells expressing antifungal PRRs in both iNeutrophils and primary human neutrophils (n = 3 biological replicates)(*p<0.05, **p<0.005, ***p<0.0005, ****p<0.0001, via a two-way ANOVA with Sidak’s multiple comparison test). (F) Representative histograms of PRR expression intensities within the CD11b+ population of all cell lines stained. (G) Quantification of the percentage of CD11b+CD15+CD16+ cells expressing antifungal PRRs in both iNeutrophils and primary human neutrophils (n = 3 biological replicates) (**p<0.005, via a two-way ANOVA with Sidak’s multiple comparison test). (H) Representative histograms of PRR expression intensities within the CD11b+CD15+CD16+ population of all cell lines stained.

To determine if the increased antifungal activity of GATA1-KO iNeutrophils is mediated by changes in the surface expression of antifungal receptors, we performed flow analysis for known human fungal pattern recognition receptors. These receptors include the β(1,3)-glucan receptors dectin-1 [33] and complement receptor 3 (CD11b/CD18 heterodimer) [10,34–36], as well as the toll-like receptors TLR2 and TLR4 [5]. Additionally, Fc-gamma receptor IIA (FcγIIR) plays an important role in the antifungal immune response against opsonized *A. fumigatus* hyphae and was assessed as well [10]. In accordance with previous reports, cell surface marker staining showed that nearly 100% of both the WT and GATA1-KO iNeutrophils expressed the general myeloid marker CD11b (Fig 2A and 2B). The GATA1-KO iNeutrophil population had significantly more cells that also expressed the neutrophil maturation markers CD15 and CD16 compared to WT iNeutrophils (Fig 2C and 2D) [28]. When analyzing CD11b+ cells, nearly 100% of both WT and GATA1-KO iNeutrophils expressed both CD18 and FcγIIR (Fig 2E and 2F). However, a significantly higher proportion of GATA1-KO iNeutrophils expressed dectin-1, TLR2 and TLR4. Interestingly, further gating on CD15+CD16+ cells showed that GATA1-KO iNeutrophils had comparable expression levels of all PRRs as primary human neutrophils in this mature population, but that WT iNeutrophils still had significantly fewer cells expressing dectin-1 (Fig 2G and 2H). Collectively, the increased maturity and PRR expression patterns on GATA1-KO iNeutrophils provides a potential mechanism for their improved antifungal defense.

### iNeutrophils shift their metabolism toward the pentose phosphate pathway in response to zymosan

**Fig 3:**
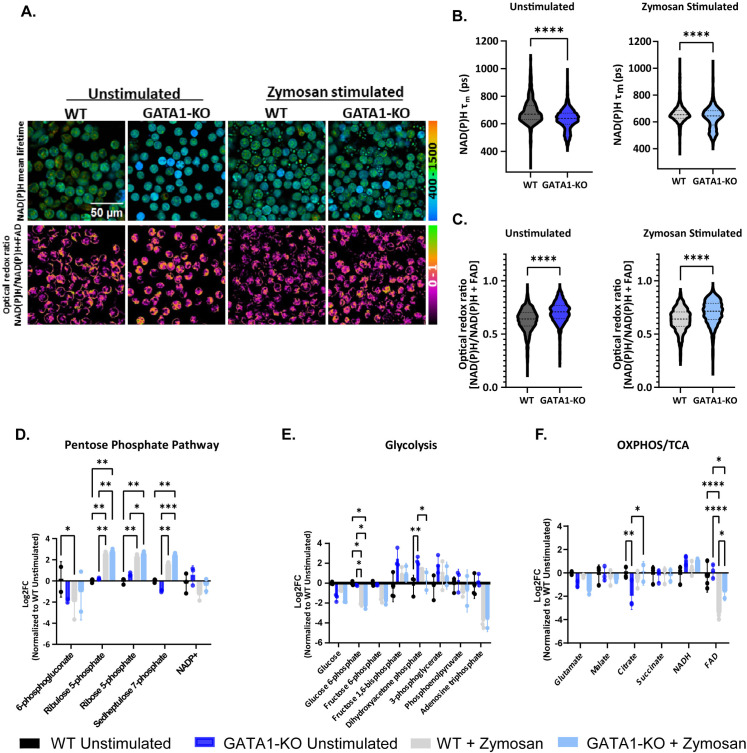
iNeutrophils rewire their metabolism following activation with zymosan. Representative image of NAD(P)H mean lifetime (top) and optical redox ratio [NAD(P)H/NAD(P)H+FAD] (bottom) used to determine the redox state of WT and GATA1-KO iNeutrophils in unstimulated and zymosan stimulated conditions. Single cell quantification of the (B) NAD(P)H mean lifetime, τ_m_, and (C) optical redox ratio of WT and GATA1-KO iNeutrophils following stimulation with 100μg/ml zymosan. (n = 3 biological replicates, 5-6 images per condition per replicate with at least 412 cells per replicate) (****p<0.0001, via Mann Whitney test). (D-F) Fold-change changes in the abundances of metabolites associated with the (D) pentose phosphate pathway (E) glycolysis and (F) the oxidative phosphorylation (OXPHOS) pathway and tricarboxcylic acid (TCA) cycle following stimulation with 100μg/ml zymosan. All samples are normalized to the WT unstimulated experimental group. (n = 3 biological replicates)(*p<0.05, **p<0.005, ***p<0.0005, ****p<0.0001, via a two-way ANOVA with Sidak’s multiple comparison test).

PRR binding to microbial pathogens initiates the antifungal activity of neutrophils during infection. Recently, metabolic rewiring towards the pentose phosphate pathway (PPP) following activation in human neutrophils has also been shown to fuel the oxidative burst associated with their antimicrobial defense [14,15]. We also found an increase in PPP in primary human neutrophils treated with zymosan, a β(1,3)-glucan rich fungal cell wall bioparticle (S2 Fig) [37]. To determine if iNeutrophils display a similar shift in metabolism in response to zymosan, we first performed single cell optical metabolic imaging. This provided single cell autofluorescence lifetime and intensity measurements of reduced nicotinamide adenine dinucleotide (phosphate) (NAD(P)H) and oxidized flavin adenine dinucleotide (FAD) [38]. The findings revealed a decrease in NAD(P)H mean lifetime and a corresponding reduced redox state (indicated by an increase in the redox ratio) of the GATA1-KO iNeutrophils in both zymosan stimulated and unstimulated experimental groups (Fig 3A–3C). The data suggest that there is likely an overall increase in both free and total NAD(P)H levels within these cells, and thus increased metabolism in the pathways associated with the production of these metabolites, such as glycolysis and the pentose phosphate pathway. To identify what pathways are specifically altered following stimulation, we next performed LC-MS-based analysis of metabolite abundances in both WT and GATA1-KO iNeutrophils. Following zymosan exposure, a significant increase in metabolites associated with the pentose phosphate pathway was observed in both iNeutrophil cell lines exposed to zymosan (Fig 3D). This change also correlated with significant reductions in ATP and fructose-6-phosphate levels. However, there were minimal changes between iNeutrophil lines for other metabolites involved in glycolysis and oxidative phosphorylation following stimulation (Fig 3E and 3F). Thus, it appears that both WT and GATA1-KO iNeutrophils upregulate the pentose phosphate pathway upon activation by zymosan, similar to primary human neutrophils (S2 Fig).

### GATA1-KO iNeutrophils display enhanced effector functions in vitro

**Fig 4:**
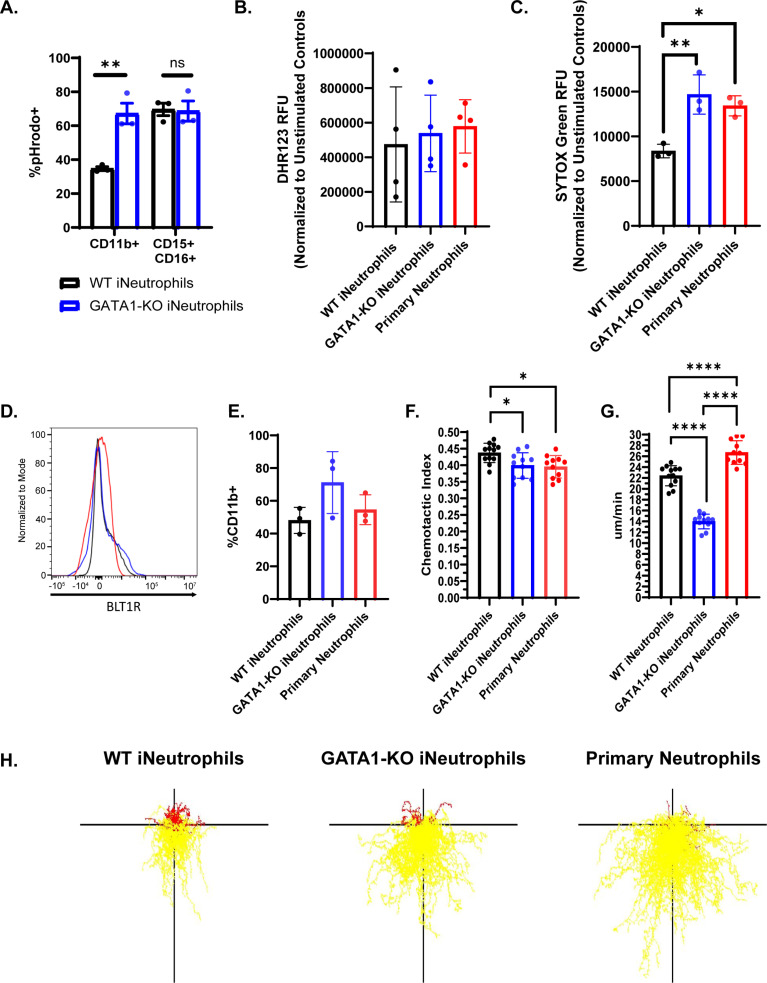
GATA1-KO iNeutrophils effectively migrate and execute effector functions *in vitro.* (A) iNeutrophil phagocytosis of pHrodo zymosan beads quantified by flow cytometry. The percent of CD11b+pHrodo+ and CD11b+CD15+CD16+pHrodo+ cells for both WT and GATA1-KO iNeutrophils are shown. (n = 3 biological replicates) (**p<0.005, via a two-way ANOVA with Sidak’s multiple comparison test). (B) Quantification of intracellular ROS production following 2 hours of coincubation with 100μg/ml zymosan. (n = 4 biological replicates)(Statistics run via one-way ANOVA with Tukey’s multiple comparison test). (C) Quantification of NET production following 6 hours of coincubation with 50ng/ml PMA. (n = 3 biological replicates)(*p<0.05, **p<0.005, via a one-way ANOVA with Tukey’s multiple comparison test). (D) Representative histogram of BLT1R expression intensity on both iNeutrophils and primary human neutrophils (colors match those associated with each cell line in Fig 4E). (E) Quantification of the number of CD11b+BLT1R+ cells in both iNeutrophils and primary human neutrophils. (n = 3 biological replicates)(Statistics run via one-way ANOVA with Tukey’s multiple comparison test). (F) iNeutrophil chemotactic index and (G) mean velocity in response to a LTB4 gradient over 45 minutes of imaging. (n = 3 biological replicates with 2-4 technical replicates per test)(*p<0.05, ****p<0.0001, via one-way ANOVA with Tukey’s multiple comparison test) (E) Representative track plots of cell migration through an LTB4 gradient. Yellow tracks indicate forward movement towards higher areas of LTB4 concentration, whereas red tracks indicate cells that moved away.

To kill fungi, neutrophils generate reactive oxygen species (ROS), release granules and antimicrobial peptides, and form neutrophil extracellular traps (NETs) [6–10]. The ability of these iNeutrophil cell lines to perform these effector functions is critical to their antifungal efficacy. The observation that GATA1-KO iNeutrophils have increased antifungal activity against *A. fumigatus* suggests that they likely have improved effector functions. Indeed, GATA1-KO iNeutrophils have already been reported to produce more NETs than WT cells [28]. However, the response to fungal cell wall epitopes had not been previously examined. Therefore, we exposed iNeutrophils to zymosan and assessed their ability to phagocytose and generate ROS following activation. GATA1-KO iNeutrophils more efficiently phagocytosed fluorescent pHrodo zymosan than WT iNeutrophils when comparing phagocytosis rates of the CD11b+ populations of both cell lines (Fig 4A). However, this difference was lost when analyzing the mature CD11b+CD15+CD16+ populations of both cells, suggesting that the enhanced phagocytosis is likely a consequence of the increased levels of mature iNeutrophils in GATA1-KO cells. No significant differences were observed when analyzing ROS production between WT iNeutrophils, GATA1-KO iNeutrophils and primary human neutrophils (Fig 4B), suggesting that all cell lines produce ROS at comparable levels following zymosan exposure. However, GATA1-KO iNeutrophils displayed increased NET formation in response to phorbol myristate acetate (PMA), at similar levels to primary human neutrophils (Fig 4C), as previously published [28]. Collectively, GATA1-KO iNeutrophils show improved phagocytosis and NETosis that likely contribute to their increased antifungal activity.

### iNeutrophils show robust chemotaxis to LTB4

Central to host defense responses is the ability of neutrophils to migrate and aggregate around invading microbes, and fungal co-incubation has shown that iNeutrophils effectively cluster around growing *A. fumigatus* hyphal structures (Fig 1). The leukotriene LTB4 is a key player in the process of neutrophil aggregation and swarming in response to fungi [32,39]. To determine if the iNeutrophil cell lines can similarly respond to LTB4 to mediate their directed migration, we performed cell surface analysis and migration assays. Cell surface marker staining for BLT1R, the high affinity LTB4 receptor [40], showed that both iNeutrophil cell lines expressed the receptor at comparable levels to primary human neutrophils (Fig 4D-4E). Using a previously published microfluidics device and live imaging of neutrophil migration [41], we found that both iNeutrophil cell lines had similar chemotactic responses to LTB4 as primary human neutrophils (Fig 4F-4H)(S2 Movie). Interestingly, GATA1-KO iNeutrophils had reduced velocity compared to WT iNeutrophils and primary neutrophils (Fig 4G). It is not clear why GATA1-KO iNeutrophils have reduced velocity, however, it is possible that the cells are more activated and adherent thereby reducing cell speed but not the chemotactic index. Nonetheless, these results show that iNeutrophils migrate effectively in response to the chemoattractant LTB4, at levels similar to primary human neutrophils.

### The integrin CD18 is necessary for *A. fumigatus* killing by iNeutrophils

To determine if iNeutrophils provide a genetically tractable model to examine pathways that mediate human neutrophil defense, we tested the effects of deleting key surface recognition components involved in neutrophil antifungal responses using GATA1-KO iNeutrophils. GATA1-KO iNeutrophils were used because they show more robust fungal killing compared to WT cells (Fig 1). Complement receptor 3 (a heterodimeric complex of CD11b/CD18) and dectin-1 are ß(1,3)-glucan receptors found on neutrophils. CR3 is the predominant human neutrophil ß(1,3)-glucan receptor that is necessary for fungal recognition and subsequent killing by primary human neutrophils [10,34–36]. However, the role that dectin-1 plays in human neutrophil antifungal immunity remains unclear [10,18–20]. Loss of dectin-1 has been shown to attenuate the antifungal activity of primary murine neutrophils [18,19], but evidence from primary human neutrophils suggest that dectin-1 is dispensable [10,20]. We have shown that iNeutrophils shift their metabolism following treatment with ß(1,3)-glucan rich zymosan particles (Fig 3), but the roles that CR3 or dectin-1 play in driving metabolic changes also remain unknown. To assess the relationship between PRR-mediated fungal killing and metabolic reprogramming, we deleted CD18 (an integral component of CR3) or dectin-1 using CRISPR-Cas9 in GATA1-KO iPSC cells (S3 and S4 Figs).

**Fig 5:**
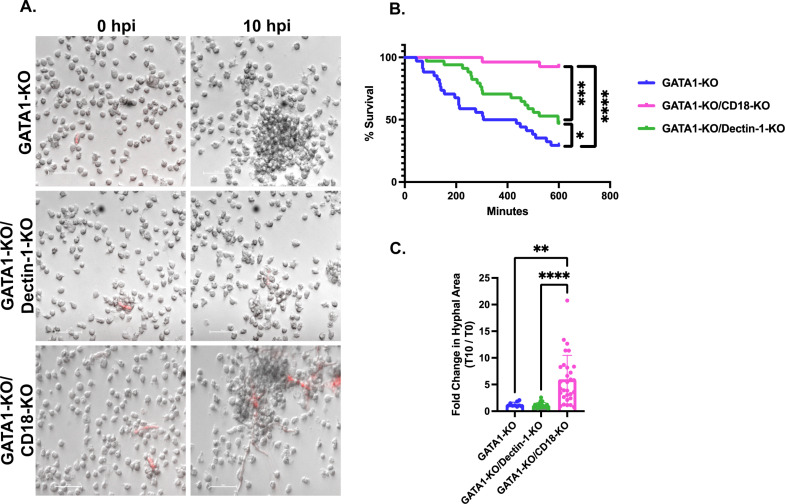
CD18 is necessary for GATA1-KO iNeutrophils to kill and attenuate the growth of *A. fumigatus.* (A) Representative images of GATA1-KO, GATA1-KO/Dectin-1-KO and GATA1-KO/CD18-KO iNeutrophils aggregating around and killing RFP expressing *A. fumigatus* germlings following 10 hours of coincubation. (Scale bar = 50μm). (B) Kaplan Meier survival curve of *A. fumigatus* following 10 hours of incubation with either GATA1-KO, GATA1-KO/Dectin-1-KO or GATA1-KO/CD18-KO iNeutrophils in RPMI media supplemented with 2% human serum. Three biological replicates were run for all experimental conditions (n = 49 fungal cells tracked for GATA1-KO iNeutrophils, n = 49 fungal cells tracked for GATA1-KO/Dectin-1-KO iNeutrophils and n = 40 fungal cells tracked for GATA1-KO/CD18-KO iNeutrophils)(*p<0.05, ***p<0.0005, ****p<0.0001 via Cox proportional hazard regression analysis). (C) Quantification of the fold change in hyphal growth of living fungal cells at 10 hpi relative to 0 hpi following incubation with either GATA1-KO, GATA1-KO/Dectin-1-KO or GATA1-KO/CD18-KO iNeutrophils. Three biological replicates were run for each condition (n = 9 fungal cells assessed for GATA1-KO treated samples, n = 22 fungal cells assessed for GATA1-KO/Dectin-1-KO treated samples and n = 30 fungal cells assessed for GATA1-KO/CD18-KO treated samples)(**p<0.005, ****p<0.0001, via one-way ANOVA with Kruskal Wallis multiple comparisons test).

Cell surface marker staining showed successful deletion of CD18 (S3A and S3B Fig). Expression of CD15 or CD16 following iNeutrophil differentiation were unchanged in GATA1-KO/CD18-KO iNeutrophils, suggesting that loss of CD18 does not affect iNeutrophil maturation (S3C and S3D Fig). CRISPR-Cas9 successfully truncated the *CLEC7a* gene encoding dectin-1 (S4A Fig), but western immunoblotting revealed reduced, but not absent, protein expression of dectin-1 in GATA1-KO/Dectin-1-KO cells (S4B Fig). Co-incubation with *A. fumigatus* showed that GATA1-KO/CD18-KO iNeutrophils were almost completely attenuated in their ability to kill fungal germlings over a 10-hour incubation period, while GATA1-KO/Dectin-1-KO iNeutrophils had only a modest reduction in fungal killing compared to GATA1-KO cells (Fig 5A and 5B). GATA1-KO/CD18-KO iNeutrophils were also unable to control hyphal development throughout this time (Fig 5C). These findings are consistent with previous studies that have demonstrated that the integrin CD18 is necessary for host defense responses in both primary mouse and human neutrophils [36,42,44–46] and demonstrates an important role for CD18 in iNeutrophil antifungal immunity. Given the modest impact that loss of dectin-1 had on fungal killing compared to CD18 deficient mutants and the residual protein present on immunoblotting of GATA1-KO/Dectin-1-KO cells, subsequent analysis was focused on the effects of CD18-KO on iNeutrophil function.

### CD18 is not required for the upregulation of the pentose phosphate pathway in response to zymosan

**Fig 6:**
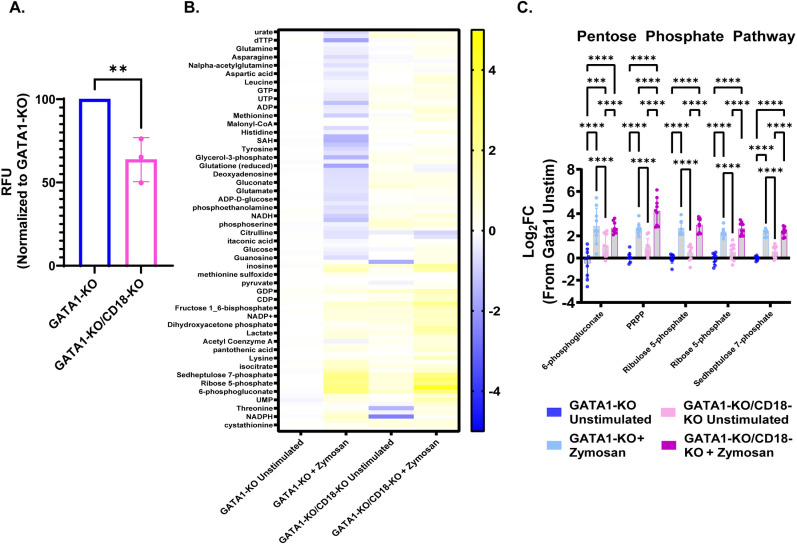
Loss of CD18 (CR3) does not impact metabolic rewiring to the pentose phosphate pathway. (A) Quantification of intracellular ROS production following 2 hours of coincubation with 100μg/ml zymosan. (n = 3 biological replicates)(**p<0.05, via student’s t-test). (B) Heatmap showing metabolite abundances in iNeutrophil lines following 2 hours of incubation with 100µg/ml zymosan. Color represents the fold-change compared to GATA1-KO unstimulated control samples. (C) Fold-change in the abundances of metabolites associated with the pentose phosphate pathway. All samples are normalized to the GATA1-KO unstimulated experimental group (n = 3 biological replicates with three technical replicates for each test)(***p<0.0005, ****p<0.0001, via a two-way ANOVA with Sidak’s multiple comparison test).

CR3 activation has been shown to induce ROS production to aid in the antifungal activity of human neutrophils [36,42]. Since the GATA1-KO/CD18-KO iNeutrophils have impaired control of *A. fumigatus* growth (Fig 5), we hypothesized that there would be attenuated ROS production following activation. To test this, we incubated both GATA1-KO and GATA1-KO/CD18-KO iNeutrophils with zymosan. Indeed, GATA1-KO/CD18-KO iNeutrophils produced significantly less ROS than the GATA1-KO parental strain following zymosan treatment (Fig 6A).

We hypothesized that part of the failure of GATA1-KO/CD18-KO iNeutrophils to kill fungi stems from an inability to shift their metabolism towards the pentose phosphate pathway following activation. To test this hypothesis, both GATA1-KO and GATA1-KO/CD18-KO iNeutrophils were stimulated with zymosan and metabolomics was performed. Overall, the metabolic profile of GATA1-KO/CD18-KO iNeutrophils showed some differences between GATA1-KO iNeutrophils in both unstimulated and zymosan stimulated conditions (Fig 6B). However, when specifically assessing metabolites generated from the pentose phosphate pathway, CD18-deficient iNeutrophils shifted their metabolism toward the PPP, similar to GATA1-KO iNeutrophils (Fig 6C). Thus, although CD18 is essential to mediate efficient *A. fumigatus* killing, it is not required for the upregulation of the pentose phosphate pathway.

### Loss of CD18 impairs iNeutrophil migration and aggregation around developing *A. fumigatus* hyphae

**Fig 7:**
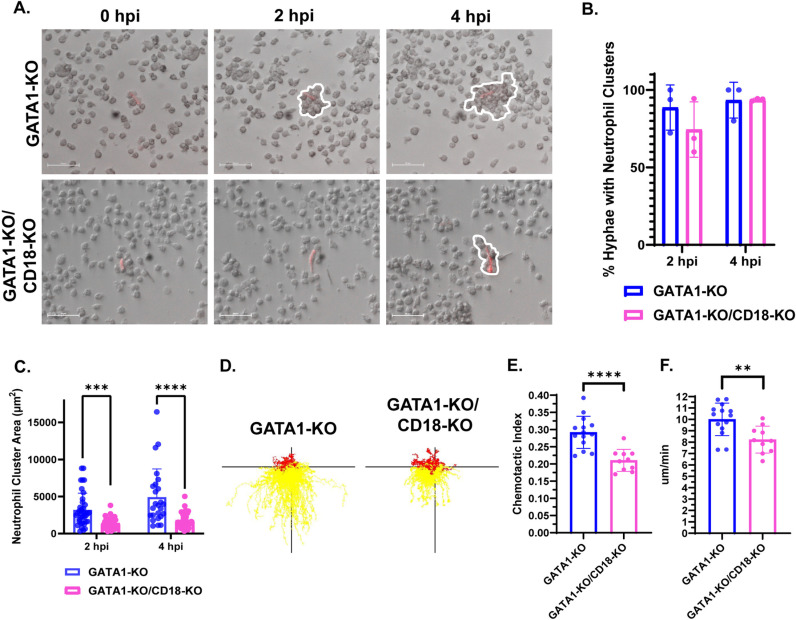
Loss of CD18 impairs the ability of GATA1-KO iNeutrophils to cluster and migrate *in vitro.* (A) Representative time-lapse images of iNeutrophils clustering around *A. fumigatus* hyphae at 2 and 4 hours post incubation. *A. fumigatus* cells are red and iNeutrophil clusters are outlined in dashed white lines. (Scale bar = 50μm). (B) Quantification of the number of fungal cells displaying neutrophil clusters around them at 2 hpi and 4 hpi. Three biological replicates were run for all samples. (n = 44 and n = 29 fungal cells assessed for GATA1-KO treated samples at 2hpi and 4hpi, respectively. n = 57 and n = 49 fungal cells assessed for GATA1-KO/CD18-KO treated samples at 2hpi and 4hpi, respectively)(Statistics were run via a two-way ANOVA with a Sidak’s multiple comparison test). (C) Quantification of the size of neutrophil clusters around *A. fumigatus* hyphae at 2 and 4 hours post incubation. (n = 38 and n = 25 neutrophil clusters assessed for GATA1-KO treated samples at 2hpi and 4hpi, respectively. n = 42 and n = 41 neutrophil clusters assessed for GATA1-KO/CD18-KO treated samples at 2hpi and 4hpi, respectively)(***p<0.0005, ****p<0.0001, via a two-way ANOVA with a Sidak’s multiple comparison test). (D) Representative track plots of GATA1-KO or GATA1-KO/CD18-KO iNeutrophils migrating through an LTB4 gradient. Yellow tracks indicate forward movement towards higher areas of LTB4 concentration, whereas red tracks indicate cells that moved away. (E) iNeutrophil chemotactic index and (F) mean velocity in response to a LTB4 gradient over 45 minutes of imaging. (n = 3 biological replicates with 2-4 technical replicates per test)(**p<0.005, ****p<0.0001, via student’s t-tests).

Human neutrophils harboring genetic mutations in *ITGB2*, the gene encoding CD18, are unable to exit circulation and migrate towards sites of infection [43]. Therefore, we hypothesized that the impaired killing observed in the GATA1-KO/CD18-KO iNeutrophils may be due to migration defects. To test this, we incubated both GATA1-KO and GATA1-KO/CD18-KO iNeutrophils with *A. fumigatus* germlings and assessed their ability to form neutrophil clusters around developing fungal cells (Fig 7A). At 2 and 4 hours post incubation, both GATA1-KO and GATA1-KO/CD18-KO iNeutrophil treated samples had comparable numbers of germlings surrounded by neutrophil clusters (Fig 7B). However, the neutrophil clusters surrounding *A. fumigatus* were significantly smaller with the GATA1-KO/CD18-KO cells (Fig 7C), suggesting impaired motility. To further assess this, we performed microfluidic chemotactic assays and found that the CD18-deficient cells had impaired chemotaxis toward LTB4 (Fig 7D-7F and S3 Movie). These findings indicate that fungal control may be impaired due to reduced migration of the CD18-KO cells.

## Discussion

Here, we show that human iPSC-derived iNeutrophils provide a genetically tractable system to understand human neutrophil antifungal defense. GATA1-KO iNeutrophils are more mature and the cells attenuate both the growth and survival of *A. fumigatus* germlings *in vitro* (Fig 1). The iNeutrophils also provide a robust system to study the metabolism of human neutrophils. Like primary human neutrophils, iNeutrophils shift their metabolism to the pentose phosphate pathway following stimulation (Figs 3 and S2). Modification of GATA1-KO iNeutrophils by deletion of the integrin CD18 demonstrated a key role for CD18 in fungal killing independent of its effects on metabolism. Taken together, our findings support the iNeutrophil system as a powerful tool to understand neutrophil biology.

Deletion of CD18 in iPSCs demonstrated an important role for CD18 in iNeutrophil motility and control of fungal growth. Studies in both humans and mice show that loss of CD18 causes increased susceptibility to bacterial and invasive fungal infections [44,45]. Additionally, *in vitro* assays using murine neutrophils harboring mutations in CD18 show that this integrin is also needed for phagocytosis, NETosis and ROS production in response to *A. fumigatus* conidia and zymosan [42,44]. CD18 deficient primary murine neutrophils are also attenuated in their ability to control the growth of *A. fumigatus* hyphae *in vitro* [36,46]. Here, we show that loss of GATA1 results in iNeutrophils that have enhanced antifungal activity compared to WT iNeutrophils (Fig 1). Deletion of CD18 in GATA1-KO iNeutrophils almost completely ablated the ability of GATA1-KO iNeutrophils to kill *A. fumigatus* germlings *in vitro*, and significantly impaired their capacity to control hyphal growth (Fig 5). Accordingly, CD18-deficient iNeutrophils were significantly impaired in their migration to fungi and chemoattractants (Fig 7) and had impaired generation of ROS following stimulation with fungal zymosan (Fig 6). Importantly, our ability to recapitulate findings from both murine and human studies highlights the efficacy of iNeutrophils as an alternative model to study neutrophil antifungal immunity. However, although GATA1-KO iNeutrophils rely on CD18 to effectively mediate their antifungal activities, both WT and GATA1-KO iNeutrophils expressed CD18 at comparable levels (Fig 2). Therefore, CD18 was likely not responsible for the increased antifungal activity of GATA1-KO iNeutrophils.

Metabolic reprogramming is an important mechanism that mediates host defense to fungi. For example, macrophages, monocytes and natural killer (NK) cells all shift their metabolism towards glycolysis following activation by fungi to mediate their antifungal functions [47–49]. Similarly, glucose transport in neutrophils has been found to be an important mediator of their effector activities in response to *C. albicans*, as loss of the glucose transporter GLUT1 in murine neutrophils attenuated their ability to phagocytose and produce ROS [50]. Recent work has also found that neutrophils further rewire their metabolism towards the pentose phosphate pathway to increase their production of NADPH, and thus increase their capacity to generate ROS (S2 Fig) [14,15]. Indeed, this shift has been shown to be important in mediating the antifungal activity of neutrophils against *A. fumigatus in vitro*. To date, only two fungal cell wall ligands have been shown to induce metabolic reprogramming in immune cells, ß-glucan and DHN-melanin [51]. Accordingly, we have shown that both WT and GATA1-KO iNeutrophils shift their metabolism towards the pentose phosphate pathway following stimulation with ß-glucan rich zymosan (Fig 3). However, loss of CR3, the primary ß-glucan receptor needed for neutrophil antifungal activity, did not impact upregulation of the pentose phosphate pathway in GATA1-KO iNeutrophils following zymosan exposure (Fig 6). This suggests a separation between metabolic shifts and some aspects of fungal growth control of iNeutrophils. iNeutrophils provide a powerful system to study human neutrophil immunometabolism and highlights the need for further research to understand receptor driven metabolic changes.

In summary, we have shown the power of GATA1-KO iNeutrophils as a model to study neutrophil-fungal interaction dynamics. The iNeutrophils show robust migration to fungi, reminiscent of a swarming response. These responses are comparable to primary human neutrophils (Fig 4), suggesting that iNeutrophils are an alternative model to study neutrophil motility in a cell system that is more like primary human cells. However, GATA1-KO cells have a reduced velocity (Fig 4). It is possible that the velocity of GATA1-KO iNeutrophils is reduced because the cells are more activated than WT iNeutrophils. Activation of primary neutrophils results in increased adhesion that can reduce migration velocity [52]. Further characterization of the GATA1-KO iNeutrophils will be needed to address the mechanism of this reduced migration.

It is interesting to consider the potential broader applications of iNeutrophils. Neutropenia leads to increased susceptibility to fungal infections [3]. Granulocyte transfusion therapy has been proposed as a potential means to circumvent these risk factors by providing neutrophils to help combat disease progression [53–55], but limited donor supply and the short lifespan and variable efficacy of primary human neutrophils has thus far limited this approach. The use of human induced pluripotent stem cell (iPSC)-derived neutrophils (iNeutrophils) provides an infinite source of genetically tractable neutrophils as a powerful alternative. Indeed, our protocol produces good manufacturing practice (GMP)-compatible iNeutrophils, opening the possibility for human treatment [26]. The challenge will be to use genetic manipulation to generate iNeutrophils that are optimized for fungal killing but have limited off-target toxicity. These studies will also advance our understanding of how primary human neutrophils mediate antifungal immunity.

## Methods

### Fungal growth media and culture conditions.

*Aspergillus fumigatus* strain CEA10 expressing a cytosolic RFP protein was used for all fungal experiments (TDGC1.2 - *ΔakuB; argB-; gpdA::RFP::argB; pyrG-; fumipyrG*) [29]. Glycerol stocks were maintained at -80 °C and struck out on solid glucose minimal media (GMM) for activation [56]. Once struck out, the plate was incubated at 37 °C in the dark for 3 days prior to the experimental start date to induce asexual conidiation. Following incubation, fungal spores were collected in 0.01% Tween 20 solution using an L-spreader and filtered through a 0.4μm mesh filter (Fischer Scientific) into a 50ml conical tube. A 5ml aliquot was then transferred to a new tube, centrifuged at 800rpm for 5 minutes and then resuspended in GMM prior to further back dilution at the start of each experiment.

### Stem cell maintenance and differentiation to iNeutrophils

Differentiation of iNeutrophils from bone marrow-derived human IISH2i-BM9 iPSCs (WiCell) was performed as previously described [57]. Briefly, iPSCs were cultured on Cultrex-coated (WiCell) tissue culture plates in mTeSR-Plus medium (STEMCELL Technologies). To begin the differentiation process, iPSC cells were passaged onto collagen-coated plates (2.4µg/mL) containing TeSR-E8 media with 10µM ROCK inhibitor Y-27632 (ROCKi; Tocris). Cells were then left to adhere to the plate for two hours while incubating at 37 °C + 5% CO_2_. Differentiation to hemogenic endothelium (starting at “Day 0”) was initiated via *ETV2* mRNA (TriLink Biotechnologies) transfection in TeSR-E8 media (STEMCELL Technologies) with the use of TransIT reagent and mRNA boost (Mirus Bio). Cells were then left to incubate for one day at 37 °C + 5% CO_2_. At day 1, the media was replaced with StemLineII media containing VEGF-165 (20ng/mL; PeproTech) and FGF2 (10ng/mL; PeproTech) to further induce differentiation into hemogenic endothelial cells. This media was then replaced at day 2 with fresh StemLineII media with VEGF-165 and FGF2. At day 3, differentiation from hemogenic endothelia to granulocyte-monocyte progenitors was initiated by changing the media to StemLineII media supplemented with FGF2 (20ng/mL), granulocyte-macrophage colony-stimulating factor (25ng/mL; PeproTech), and UM171 (50nM; Xcess Biosciences). Cells were then left to incubate at 37 °C + 5% CO_2_ before topping off the media at day 7 with an equal volume of fresh StemLineII media supplemented FGF2, granulocyte-macrophage colony-stimulating factor and UM171. At day 11, non-adherent progenitor cells were collected and used for iNeutrophil differentiation. Following collection, progenitor cells were cultured in StemSpan SFEM II medium (STEMCELL Technologies), supplemented with GlutaMAX 100× (1×; Thermo Fisher Scientific), ExCyte 0.2% (Merck Millipore), human granulocyte colony-stimulating factor (150ng/mL; PeproTech), and Am580 retinoic acid agonist (2.5μM; STEMCELL Technologies) at 0.8-1 × 10^6^ cells/mL density. After 4 days, suspensions were topped off with equal volumes of StemSpan SFEM II medium supplemented with GlutaMAX, ExCyte, human granulocyte colony-stimulating factor and Am580. iNeutrophils were then collected for experimentation between days 5-7 following initial progenitor harvesting.

### Generation of mutant iPSC cell lines

CRISPR-Cas9 was used as previously described to generate GATA1, CD18 and dectin-1 deficient IISH2i-BM9 iPSCs [23]. To generate a GATA1-KO mutant, two guide RNAs (sgRNAs) were designed with the use of Synthego Guide Design to target exon 2 in the coding sequence of *GATA1* (gRNA1 – 5’ CCAUGGAGUUCCCUGGCCUG 3’; gRNA2 – 5’ CAGGAUCCACAAACUGGGGG 3’). Prior to nucleofection, WT iPSC cells were lifted using TrypLE Select (Life Technologies) and singularized by pipetting. Then, 5µg of Cas9 protein (PNA Bio) and 2.5µg of each of the sgRNAs were incubated together for at least 10 minutes at room temperature before nucleofection of iPSC cells occurred using a Human Stem Cell Nucleofector Kit 2 (Lonza). Cells were then plated at 25 cell/cm^2^ on 2x Cultrex-coated plates in mTeSR-Plus media with 1× CloneR supplement (STEMCELL Technologies). Plates were incubated for 2 days before changing the media to mTeSR-Plus. Incubation continued until individual colonies with smooth edges were visible. Then, individual colonies were picked and further expanded in individual wells of a 1x Cultrex-coated 12-well plate. Following expansion, genomic DNA was extracted from individual clones and screened for the loss of a 32 basepair fragment in exon 2 of *GATA1* via polymerase chain reaction (PCR) using forward (5’ ATGGAGACTGAGGTGATGGAGTGG 3’) and reverse (5’ TGCAGCGGTGGCTGTGCTC 3’) screening primers.

To generate a GATA1-KO/CD18-KO double mutant, sgRNAs targeting exon 4 in the coding sequence of *ITGB2* (gRNA1 – 5’ CGAAUGGAGUCAGGAUCCCC 3’; gRNA2 – 5’ GCUGCUCAUGAGGGGCUGUG 3’) were designed using Synthego Guide Designer. Nucleofection into the GATA1-KO iPSC parental strain and subsequent expansion of single colonies was then performed as described above. Mutants harboring truncations within *ITGB2* were then confirmed via PCR using forward (5’ CAGGTCCCGCAGTGTG 3’) and reverse (5’ GTTTCAGCGAGGCTTGTG 3’) screening primers. Mutants were identified by loss of a 52 basepair fragment in the PCR product and confirmed by flow cytometry staining for CD18 following differentiation to iNeutrophils.

To generate a GATA1-KO/Dectin-1-KO double mutant, sgRNAs targeting exon 2 in the coding sequence of *CLEC7a* (gRNA1 – 5’ CCAAUUAGGAGGACAAGGGC 3’; gRNA2 – 5’ CAGCAUGUCACUAAAUUCCU 3’) were designed using Synthego Guide Designer. Nucleofection into the GATA1-KO iPSC parental strain and subsequent expansion of single colonies was then performed as described above. Mutants harboring truncations within *CLEC7a* were then confirmed via PCR using forward (5’ GAGTGAATTGGTATGCTATGAACC 3’) and reverse (5’ GCTGTCTATCTTTAGGAGATTAGAGC 3’) screening primers. Mutants were identified by loss of a 63 basepair fragment in the PCR product and confirmed by western blot analysis for the dectin-1 protein.

### Primary human neutrophil collection

Neutrophils were harvested from blood collected from healthy volunteers under a University of Wisconsin–Madison Minimal Risk Research Institutional Review Board–approved protocol (ID: 2017–0032). Formal written consent was obtained from donors prior to blood draw. Immediately following blood collection, neutrophils were isolated via the use of a MACSxpress negative antibody collection kit (Miltenyi Biotec) per the manufacturer’s instructions. Following isolation, neutrophils were resuspended in 1x phosphate buffered saline (PBS)(Gibco) and subsequently used for different experiments.

### iNeutrophil fungal killing assays

To assess neutrophil/fungal interactions, *A. fumigatus* strain CEA10-RFP (TDGC1.2) was grown as described above. Following spore collection, spores were quantified via hemocytometer and diluted to a final concentration of 4 x 10^3^ cells/ml in GMM. For terminal assays, in which microscopy images were only taken at the start of the experiment and 4 hours post-incubation, 100μl of the spore suspension was added to the appropriate number of wells in a 96-well clear, flat bottom plate previously coated in 10μg/ml fibrinogen and left to incubate at 37 °C + 5% CO_2_ for 8 hours, or until germlings appeared. Roughly two hours prior to the end of the spore incubation, primary human neutrophils were collected as previously described and diluted to a cell concentration of 4 x 10^5^ cells/ml in RPMI + 2% heat-inactivated fetal bovine serum (FBS) or 2% pooled human serum. Approximately 20 minutes prior to the end of the spore incubation, iNeutrophils were collected and diluted to a cell concentration of 4 x 10^5^ cells/ml in RPMI + 2% heat-inactivated fetal bovine serum (FBS) or 2% intact pooled human serum. After fungal germling development, the media was removed from all wells in the 96-well plate and replaced with 100μl of the appropriate neutrophil suspension to yield a neutrophil:germling ratio of 100:1. The plate was then immediately taken to a Nikon Eclipse Ti inverted microscope with a preheated chamber set to 37 °C and initial images, denoted as time 0 (T0), were taken for all wells. All locations imaged in the plate were tracked in the NIS Element software package connected to the microscope. Following image acquisition, the plate was incubated for 4 hours at 37 °C + 5% CO_2_. After 4 hours (T4), the plate was removed and imaged at the same locations captured at the start of the experiment. Images were then analyzed for the presence/absence of red hyphal fluorescence between T4 and T0, and for the formation of neutrophil clusters around hyphal cells that were present at the T4 time point. Additionally, hyphal growth was quantified using Fiji ImageJ (National Institute of Health, Bethesda, MD, USA) by measuring fungal size for all viable fungi at T4 and T0 to determine their fold-change in growth over the incubation period. For all conditions, three biological replicates with three technical replicates were performed using three separate donors for primary human neutrophils or three separate pools of differentiated iNeutrophils. Statistical differences were assessed via a two-way ANOVA with Sidak’s multiple comparison test (GraphPad Prism, v7.0c software).

To further assess the kinetics of fungal killing, time lapse microscopy was performed on (i)neutrophil-fungi cocultures. Following spore collection, 1ml of a 4 x 10^3^ spore suspension in GMM was added to the appropriate number of wells of a 24-well plate previously coated with 10μg/ml fibrinogen. The plate was then left to incubate at 37 °C + 5% CO_2_ for 8 hours, or until germlings appeared. During incubation, iNeutrophils and primary human neutrophils were collected and diluted to a concentration of 8 x 10^5^ cells/ml as previously described. Following germling development, GMM media was removed from all wells and replaced with 500μl of the appropriate (i)neutrophil suspension to yield a neutrophil:germling ratio of 100:1. Neutrophil-fungal interactions were then imaged every 3 minutes for 10-12 hours on a Nikon Eclipse TE300 inverted fluorescent microscope (Nikon) with a 20× objective. Environmental controls were set to 37 °C with 5% CO_2_. Videos were compiled using the NIS Element software package connected to the microscope and ImageJ software. All experiments were performed using three biological replicates, as defined by three separate donors for primary human neutrophils or three separate pools of differentiated iNeutrophils. Fungal killing was tracked by marking the time point in which fungal RFP signal was lost in all experiments. Statistical tests to measure differences in survival were performed in RStudio using Cox proportional hazard regression analysis with experimental condition included as a group variable, as previously described [58]. Hyphal growth was quantified using Fiji ImageJ by measuring fungal size for all viable fungi at the start of the experiment (T0) and end (T10) to determine their fold-change in growth over the incubation period. Data normality was then assessed with the use of a D’Agostino-Pearson omnibus normality test and statistical significance was determined by a one-way ANOVA with a non-parametric Kruskal-Wallis test (GraphPad Prism, v7.0c software). Neutrophil clusters were identified as previously described [14,23]. Briefly, aggregates of five or more neutrophils around a fungal germling, with an associated change in their morphology from circular to irregular, were defined as a cluster. Neutrophil cluster sizes were measured using Fiji ImageJ at 2 and 4 hours post incubation to assess neutrophil activity as well. Statistical differences were determined via a two-way ANOVA with Sidak’s multiple comparison test (GraphPad Prism, v7.0c software).

### Cell staining and flow cytometry

iNeutrophils and Neutrophils were stained in PBS + 1% human serum albumin (HAS) + Brilliant Buffer (Thermo Fisher Scientific) and Human TruStain FcX Fc Receptor Blocking Solution (BioLegend). Following staining, cells were fixed in 2% paraformaldehyde and then analyzed using an Aurora Cytometer (CytekBio). Antibodies used in this study can be found in S1 Table. Data were then analyzed using FlowJo software (v10.8.1; TreeStar). Forward and side scatter parameters were used to differentiate single cells, and Zombie NIR dye was used to assess viability. Within live cells, positive signal for CD11b was used to denote myeloid cells, and mature (i)Neutrophils within that population were further classified by their expression of both CD15 and CD16. The number of cells expressing, and the intensity of their expression profiles, for the antifungal PRRs dectin-1, TLR2, TLR4, CD32 and CD18 within CD11b+ and CD11b+CD15+CD16+ populations were recorded for all cell lines tested.

### Western blot analyses

For western blots, iNeutrophils were washed with 1x PBS and treated with lysis buffer (50 mM Tris at pH 7.6, 500 mM NaCl, 0.1% SDS, 0.5% deoxycholate, 1% Triton X-100, 0.5 mM MgCl2, 1:1000 Halt protease and Phosphatase inhibitor cocktail) on ice. Cells were then sonicated with 20% amplitude for 3x 5 seconds. Lysates were then clarified by centrifugation at 20,000×g at 4 °C for 15 minutes. Protein concentrations were determined using the Pierce BCA Protein Assay (Thermo Fisher Scientific; 23225). Equal amounts of total protein were denatured in SDS sample buffer, resolved on 4–12% Bolt gradient SDS–PAGE gels and transferred to nitrocellulose. Membranes were probed using goat-anti-human Dectin-1/CLEC7A (Clone: AF1859-SP; R&D Systems) and mouse-anti-human actin AC15 (Clone: AF441; Sigma) primary antibodies and Alexa Fluor 680 goat-anti-mouse (Clone: A21057; Invitrogen) and IRDye 800CW donkey-anti-goat (Clone: 926-32214; LI-COR) secondary antibodies. Western blots were imaged with an Odyssey Infrared Imaging System (LI-COR Biosciences).

### Single cell optical metabolic imaging

For optical metabolic imaging, iNeutrophils were plated at 200,000 cells along with the 100μg/mL of zymosan on 35mm glass-bottom dishes (MatTek) about 15 minutes prior to imaging. The dishes were coated with 5µg/ml P-selectin (Bio-Techne) by incubating overnight at 4^o^C. The cells were housed in a stage top incubator (Tokai Hit) at 37^o^C and under 5% CO_2_ during the entire duration of imaging.

Optical metabolic imaging was performed on the Ultima Multiphoton Imaging System (Bruker) consisting of an inverted microscope (TI-E, Nikon) coupled to an ultrafast tunable laser source (Insight DS+, Spectra Physics Inc) and time-correlated single-photon counting electronics (SPC -150, Becker & Hickl GmbH) for lifetime measurements. NAD(P)H and FAD were sequentially excited at 750nm and 890nm while their emission was collected using bandpass filters of 460/80nm and 500/100nm, respectively, on GaAsP photomultiplier tubes (H7422P-40, Hamamatsu, Japan). The cells were illuminated using a 40x objective lens (W.I./1.15 NA/Nikon PlanApo). Pixel dwell time of 4.8µs and frame integration of 60 seconds was used with a 2x zoom of field of view (~0.9 mm^2^). The second harmonic generation (SHG) signal of urea crystals (Sigma-Aldrich) excited at 890nm was collected daily for the instrument response function (IRF).

Fluorescence lifetime data analysis was performed using SPCImage software (Becker & Hickl). To increase photon counts at each pixel, a 3x3 binning containing 9 surrounding pixels was applied. To compute the lifetimes, Weighted Least Squares algorithm was used which entailed an iterative parameter optimization to obtain the lowest sum of the squared differences between model and data. Both NAD(P)H and FAD exist in 2 states depending on their binding status such that NAD(P)H has a shorter lifetime while FAD has a longer lifetime in their free states compared to their respective bound state. Thus the pixel-wise NAD(P)H and FAD decay curves were fit to a biexponential model [I(t) = α_1_ × exp (−τ/τ_1_) + α_2_ × exp(−τ/τ_2_) + C)] convolved with the system IRF such that I(t) is the fluorescence intensity measured at time t, α_1_, α_2_ are the fractional contributions and τ_1_, τ_2_ denote the short and long lifetime components, respectively. C accounts for background light. The goodness of the fit was checked using a reduced chi-squared value<1.0. The mean lifetime is the weighted average of the free and bound lifetimes (τ_m_ = α_1_ × τ_1_ + α_1_ × τ_2_) and is calculated for each pixel. The area under the fluorescence decay curve at each pixel was integrated to generate the intensity of NAD(P)H and FAD. The optical redox ratio at each pixel is computed as the intensity ratio [NAD(P)H/ (NAD(P)H + FAD)]. For generating single cell data, single cell masks were computed from the NAD(P)H intensity image using Cellpose 2.0 (model = cyto 2, radius = 30) [59] followed by manual checks and edits on napari [60]. Pixels within cell masks that contained zymosan were manually identified by their shape and size and were excluded from analysis. A custom python library, Cell Analysis Tools [61],was used for processing and extracting the single cell data.

### Metabolomic analysis

To assess metabolite abundances following zymosan exposure, 2 x 10^6^ iNeutrophils were collected and transferred to a 1.5ml microcentrifuge tube. Cells were then washed once with PBS and resuspended in 500μl of StemSpan SFEM II medium (STEMCELL Technologies), supplemented with GlutaMAX 100x, ExCyte 0.2%, human granulocyte colony-stimulating factor (150 ng/mL), and Am580 retinoic acid agonist (2.5 μM). Following resuspension, 500μl of zymosan (Invivogen) supplemented media was added to the appropriate tubes to make a final working concentration of 100μg/ml. Similarly, media without zymosan was added to designated tubes to serve as an unstimulated control. Tubes were then left to incubate at 37 °C + 5% CO_2_ for two hours, with gentle mixing via pipetting occurring every 40 minutes throughout the incubation process. Following incubation, cells were centrifuged at 200xg for 5 minutes, the supernatant was removed and the cell pellet subsequently washed with 1ml of PBS. After washing, 500μl of an ice cold 80:20 methanol:water (MeOH:H_2_O) solution was added to each tube, vortexed and then placed in a -80 °C freezer for 30 minutes. Following incubation, tubes were vortexed again and spun at 13,000rpm for 5 minutes at 4 °C. The supernatant was transferred to a new microcentrifuge tube over dry ice. 150μl of an ice cold 80:20 MeOH:H_2_O solution was then added to the tube containing the original pellet, and the tube was vortexed, centrifuged and the supernatant was transferred to the same tube corresponding to each sample as was done before. For each condition tested, extraction of three biological replicates with three technical replicates each was performed for analysis.

Metabolites were measured using a Thermo Q-Exactive mass spectrometer coupled to a Vanquish Horizon UHPLC. Data were collected with Xcalibur 4.0 software (Thermo) and peak integration was performed using Maven [62,63]. The data was collected on a full scan negative mode. The metabolites identified were based on exact m/z and retention times that were determined with chemical standards. After isolation in -80 °C 80:20 MeOH:H_2_O, metabolite extracts were dried under nitrogen stream. Samples were resuspended in LC-MS grade water and separated on a 2.1 × 100mm, 1.7μM Acquity UPLC BEH C18 Column (Waters). The solvents used were A: 97:3 H_2_O:MeOH (v:v), 10mM tributylamine, 9 mM acetate, pH 8.2 and B: 100% MeOH. The gradient was 0 min, 95% A; 2.5 min, 95% A; 17 min, 5% A; 21 min, 5% A; 21.5 min 95% A. The flow rate was 0.2 ml/min and the column temperature was 30 °C. Setting for the ion source were; 10 aux gas flow rate, 35 sheath gas flow rate, 2 sweep gas flow rate, 3.2kV spray voltage, 320 °C capillary temperature and 300 °C heater temperature.

### Phagocytosis assays

The phagocytic capabilities of (i)Neutrophils were assessed with the use of pHrodo Green zymosan BioParticles (Invitrogen) via the manufacturer’s instructions. Briefly, the BioParticles were opsonized with 30% pooled human serum (#MP092930149; MP Biomedicals) for 30 minutes at 37 °C then washed 3 times in PBS. One million cells were resuspended in 80µL of StemSpan SFEM II medium, then20 µL of opsonized beads were added, giving a 100:1 multiplicity of infection. Cells and beads were incubated in an Eppendorf tube for 1 hour at 37 °C, then stopped by addition of ice-cold PBS. While keeping tubes on ice, cells were stained with neutrophil lineage markers (S1 Table), then fixed with 2% PFA before flow cytometry analysis on the Aurora Cytometer (CytekBio).

### Reactive oxygen species quantification

Intracellular peroxynitrate production was assessed via DHR123 (Invitrogen) staining. Briefly, 100μl of 1 x 10^5^ cells of either iNeutrophils or primary human neutrophils in RPMI + 2% FBS + 5μg/mL DHR123 was added to wells of a black 96-well clear-bottom plate previously coated with 10μg/mL fibrinogen (Sigma). Cells were then left to incubate for 1 hour at 37 °C + 5% CO_2_ before a working concentration of 100μg/mL of zymosan was added to the appropriate wells. Control wells containing a corresponding volume of media without zymosan were generated as well. Following zymosan addition, plates were incubated in the dark at 37 °C + 5% CO_2_ before their fluorescent signal was measured at 2 hours post-incubation at 485/535nm using the Victor3V microplate reader (PerkinElmer). Background signal from unstimulated cells were subtracted from zymosan stimulated samples for analysis. Three biological replicates were run for all cell lines tested, and statistical analysis was performed using either a student’s t-test or a one-way ANOVA with a Tukey’s multiple comparison analysis (GraphPad Prism, v7.0c software).

### NETosis assays

NET release was quantified by measuring extracellular DNA via SYTOX green staining as previously described [29]. To achieve this, 2 x 10^5^ cells in RPMI + 2% FBS media were added to wells of a black, clear-bottom 96-well plate previously coated with 10μg/mL fibrinogen. PMA was then added to a working concentration of 100nM to the appropriate wells. Control wells containing a corresponding volume of media without PMA were generated as well. The plate was then left to incubate in the dark at 37 °C + 5% CO_2_ for 6 hours before adding SYTOX Green (Thermofisher) to a working concentration on 375nM to all wells. Fluorescence (485nm/535nm, 0.1s) was measured 15 minutes after addition of SYTOX Green using a Victor3V plate reader (PerkinElmer). Background signal from unstimulated cells were subtracted from PMA stimulated samples for analysis. Three biological replicates were run for all cell lines tested, and statistical analysis was performed using a one-way ANOVA with a Tukey’s multiple comparison analysis (GraphPad Prism, v7.0c software).

### PrestoBlue staining for fungal viability

PrestoBlue staining was used to assess fungal growth following coincubation with (i)neutrophils. Following collection, spores were quantified via hemocytometer and diluted to a final concentration of 4 x 10^3^ cells/ml in GMM. 100μl of the spore suspension was then added to the appropriate number of wells in a 96-well clear, flat bottom plate previously coated in 10μg/ml fibrinogen and left to incubate at 37 °C + 5% CO_2_ for 8 hours, or until germlings appeared. Following germling appearance, the media was removed and 100μl of a 4 x 10^5^ cells/ml solution of either iNeutrophils or primary human neutrophils in RPMI + 2% FBS or 2% human serum were added to each well. Additionally, wells containing growth media with fungus alone were created to serve as a control to quantify fungal attenuation. All wells were then topped off with an additional 100μl of the appropriate media to prevent them from drying out during incubation and then plates were left for 24 hours at 37 °C + 5% CO_2_. Once the incubation was complete, the plate was centrifuged at 3500rpm for 5 minutes to pellet cellular material to the bottom of the plate. The media was then gently removed, replaced with 200ul of a neutrophil lysis solution (basic H_2_O (pH 11) + 100 100μg/ml DNAse I) and then left to incubate for 30 minutes at 37 °C. This process was then repeated two additional times or until (i)neutrophils were visibly lysed under brightfield microscopy. Following (i)neutrophil lysis, the media was replaced with GMM + PrestoBlue viability reagent (1:10 dilution, ThermoFisher) and the plate was left to incubate for 3 hours at 37 °C. Following incubation, media was removed and added to fresh wells that did not contain fungi and fluorescence was measured at 555/590nm using a PerkinElmer Victor3V plate reader. Three biological replicates were run for all cell lines tested in each condition, and statistical analysis was performed using a two-way ANOVA with a Sidak’s multiple comparison test (GraphPad Prism, v7.0c software).

### LTB4 chemotaxis assays

Chemotaxis was assessed using a microfluidic device as described previously [41]. In brief, polydimethylsiloxane (PDMS) devices were plasma treated and adhered to glass coverslips. Devices were coated with 10μg/mL fibrinogen + 1ug/ml fibronecin + 1.2ug/ml Collogen IV (Sigma) in PBS for 30 min at 37 °C + 5% CO_2_ or at 4 °C overnight. The devices were then blocked with 2% bovine serum albumin (BSA)-PBS for 30 min at 37 °C + 5% CO_2_, and then washed twice with PBS. Cells were stained with Calcein AM (Molecular Probes) in PBS for 20 min at 37 °C + 5% CO_2_, washed once with PBS and then resuspended in imaging media (0.25% StemSpan-II, 0.5% FBS in PBS). Cells were seeded at 8 × 10^6^ cells/mL and allowed to settle for 10 minutes before addition of chemoattractant. 3μl of 3μM LTB_4_ was loaded into the input port of the microfluidic device. Cells were imaged every 30 seconds for 60 minutes on a Nikon Eclipse TE300 inverted fluorescent microscope with a 10× objective and an automated stage using MetaMorph software (Molecular Devices). Automated cell tracking analysis was done using JEX software [64] to calculate chemotactic index and velocity and to generate representative rose plots for migration patterns.

## Supporting information

S1 Fig Confirmation of 32bp deletion in the ORF of GATA1. (A) Agarose gel showing a shift in the GATA1 gene following CRISPR-Cas9 mediated deletion of a 32 bp fragment in exon 2 of the coding sequence for the gene. (B) Sanger sequencing results confirming loss of the 32 bp fragment in exon 2 of the GATA1-KO mutant.
(TIF)

S2 Fig Primary human neutrophils upregulate the pentose phosphate pathway following activation by fungal zymosan. Fold-change in the abundances of metabolites associated with the pentose phosphate pathway. All samples are normalized to the unstimulated experimental group (n = 2 biological replicates from 2 independent donors).
(TIF)

S3 Fig Loss of CD18 does not impact surface expression of CD15 and CD16. (A) Agarose gel showing a shift in the ITGB2 gene following CRISPR-Cas9 mediated deletion of a 52bp fragment in exon 4 of the coding sequence of the gene. (B) Representative histograms of surface marker staining for CD18 in GATA1-KO and GATA1-KO/CD18-KO iNeutrophils. Fluorescence minus one (FMO) PE is indicative of a negative control for CD18 staining where the anti-CD18-PE antibody was not added to the cells. (C) Quantification of the number of CD15+CD16+ cells within the live population of iNeutrophils. (D) Representative scatter plots of CD15 and CD16 expression intensities for GATA1-KO (blue) and GATA1-KO/CD18-KO (pink) iNeutrophils.
(TIF)

S4 Fig Confirmation of dectin-1 deletion in GATA1-KO iNeutrophils. (A) Agarose gel showing a shift in the CLEC7a gene following CRISPR-Cas9 mediated deletion of a 63 bp fragment in exon 2 of the coding sequence of the gene. (B) Representative western blot showing dectin-1 protein levels in WT and KO iNeutrophils.
(TIF)

S1 Movie GATA1-KO iNeutrophils aggregate around and kill A. fumigatus in vitro. Representative movie of GATA1-KO iNeutrophils interacting with and killing A.fumigatus germlings (red) during coincubation in RPMI + 2% human serum. Images were taken every 3 minutes over a 12-hour incubation period. Scale bar is 50µm. Movie is displayed as 7 frames/second.
(AVI)

S2 Movie iNeutrophils migrate efficiently in response to the chemoattractant LTB4. Representative fluorescent movies of WT (left) and GATA1-KO (center) and primary human (right) (i)Neutrophils migrating towards an LTB4 gradient at the bottom of the image. iNeutrophils are stained with calcein and tracked using the fluorescent images. Cell tracks of quantified cells are overlaid and appear yellow. Scale bar is 100µm. Images were taken every 30 seconds for 45 minutes. Movie is displayed as 7 frames/second.
(AVI)

S3 Movie Loss of CD18 impairs GATA1-KO iNeutrophil migration efficiently to the LTB4. Representative fluorescent movies of GATA1-KO (left) and GATA1-KO/CD18-KO (right) iNeutrophils migrating towards an LTB4 gradient at the bottom of the image. iNeutrophils are stained with calcein and tracked using the fluorescent images. Cell tracks of quantified cells are overlaid and appear yellow. Note the reduced track length of GATA1-KO/CD18-KO cells compared to their parental control. Scale bar is 100µm. Images were taken every 30 seconds for 45 minutes. Movie is displayed as 7 frames/second.
(AVI)

S1 Table Antibodies used in this study. (DOCX)

## References

[1] Bongomin F, Gago S, Oladele RO, Denning DW. Global and multi-national prevalence of fungal diseases-estimate precision. J Fungi (Basel). 2017;3(4):57. doi: 10.3390/jof3040057 29371573 PMC5753159

[2] Benedict K, Whitham HK, Jackson BR. Economic burden of fungal diseases in the United States. Open Forum Infect Dis. 2022;9(4):ofac097. doi: 10.1093/ofid/ofac097 35350173 PMC8946773

[3] Segal BH, Walsh TJ. Current approaches to diagnosis and treatment of invasive aspergillosis. Am J Respir Crit Care Med. 2006;173(7):707–17. doi: 10.1164/rccm.200505-727SO 16387806

[4] Margalit A, Kavanagh K. The innate immune response to aspergillus fumigatus at the alveolar surface. FEMS Microbiol Rev. 2015;39(5):670–87. doi: 10.1093/femsre/fuv018 25934117

[5] Bojang E, Ghuman H, Kumwenda P, Hall RA. Immune sensing of candida albicans. J Fungi (Basel). 2021;7(2):119. doi: 10.3390/jof7020119 33562068 PMC7914548

[6] Tkalcevic J, Novelli M, Phylactides M, Iredale JP, Segal AW, Roes J. Impaired immunity and enhanced resistance to endotoxin in the absence of neutrophil elastase and cathepsin G. Immunity. 2000;12(2):201–10. doi: 10.1016/s1074-7613(00)80173-9 10714686

[7] Orsi N. The antimicrobial activity of lactoferrin: current status and perspectives. Biometals. 2004;17(3):189–96. doi: 10.1023/b:biom.0000027691.86757.e2 15222464

[8] Zarember KA, Sugui JA, Chang YC, Kwon-Chung KJ, Gallin JI. Human polymorphonuclear leukocytes inhibit Aspergillus fumigatus conidial growth by lactoferrin-mediated iron depletion. J Immunol. 2007;178(10):6367–73. doi: 10.4049/jimmunol.178.10.6367 17475866

[9] McCormick A, Heesemann L, Wagener J, Marcos V, Hartl D, Loeffler J, et al. NETs formed by human neutrophils inhibit growth of the pathogenic mold Aspergillus fumigatus. Microbes Infect. 2010;12(12–13):928–36. doi: 10.1016/j.micinf.2010.06.009 20603224

[10] Gazendam RP, van Hamme JL, Tool ATJ, Hoogenboezem M, van den Berg JM, Prins JM, et al. Human neutrophils use different mechanisms to kill aspergillus fumigatus conidia and hyphae: evidence from phagocyte defects. J Immunol. 2016;196(3):1272–83. doi: 10.4049/jimmunol.1501811 26718340

[11] Fuchs TA, Abed U, Goosmann C, Hurwitz R, Schulze I, Wahn V, et al. Novel cell death program leads to neutrophil extracellular traps. J Cell Biol. 2007;176(2):231–41. doi: 10.1083/jcb.200606027 17210947 PMC2063942

[12] Reeves EP, Lu H, Jacobs HL, Messina CGM, Bolsover S, Gabella G, et al. Killing activity of neutrophils is mediated through activation of proteases by K+ flux. Nature. 2002;416(6878):291–7. doi: 10.1038/416291a 11907569

[13] Winterbourn CC, Kettle AJ, Hampton MB. Reactive oxygen species and neutrophil function. Annu Rev Biochem. 2016;85:765–92. doi: 10.1146/annurev-biochem-060815-014442 27050287

[14] Britt EC, Qing X, Votava JA, Lika J, Wagner AS, Shen S, et al. Activation induces shift in nutrient utilization that differentially impacts cell functions in human neutrophils. Proc Natl Acad Sci U S A. 2024;121(39):e2321212121. doi: 10.1073/pnas.2321212121 39284072 PMC11441510

[15] Britt EC, Lika J, Giese MA, Schoen TJ, Seim GL, Huang Z, et al. Switching to the cyclic pentose phosphate pathway powers the oxidative burst in activated neutrophils. Nat Metab. 2022;4(3):389–403. doi: 10.1038/s42255-022-00550-8 35347316 PMC8964420

[16] Lahoz-Beneytez J, Elemans M, Zhang Y, Ahmed R, Salam A, Block M, et al. Human neutrophil kinetics: modeling of stable isotope labeling data supports short blood neutrophil half-lives. Blood. 2016;127(26):3431–8. doi: 10.1182/blood-2016-03-700336 27136946 PMC4929930

[17] Zschaler J, Schlorke D, Arnhold J. Differences in innate immune response between man and mouse. Crit Rev Immunol. 2014;34(5):433–54. 25404048

[18] Werner JL, Metz AE, Horn D, Schoeb TR, Hewitt MM, Schwiebert LM, et al. Requisite role for the dectin-1 beta-glucan receptor in pulmonary defense against Aspergillus fumigatus. J Immunol. 2009;182(8):4938–46. doi: 10.4049/jimmunol.0804250 19342673 PMC3434356

[19] Werner JL, Gessner MA, Lilly LM, Nelson MP, Metz AE, Horn D, et al. Neutrophils produce interleukin 17A (IL-17A) in a dectin-1- and IL-23-dependent manner during invasive fungal infection. Infect Immun. 2011;79(10):3966–77. doi: 10.1128/IAI.05493-11 21807912 PMC3187263

[20] Gazendam RP, van Hamme JL, Tool ATJ, van Houdt M, Verkuijlen PJJH, Herbst M, et al. Two independent killing mechanisms of Candida albicans by human neutrophils: evidence from innate immunity defects. Blood. 2014;124(4):590–7. doi: 10.1182/blood-2014-01-551473 24948657

[21] Rafiq M, Rivieccio F, Zimmermann A-K, Visser C, Bruch A, Krüger T, et al. PLB-985 neutrophil-like cells as a model to study aspergillus fumigatus pathogenesis. mSphere. 2022;7(1):e0094021. doi: 10.1128/msphere.00940-21 34986319 PMC8730815

[22] Blanter M, Gouwy M, Struyf S. Studying Neutrophil Function in vitro: Cell Models and Environmental Factors. J Inflamm Res. 2021;14:141–62. doi: 10.2147/JIR.S284941 33505167 PMC7829132

[23] Giese MA, Bennin DA, Schoen TJ, Peterson AN, Schrope JH, Brand J, et al. PTP1B phosphatase dampens iPSC-derived neutrophil motility and antimicrobial function. J Leukoc Biol. 2024;116(1):118–31. doi: 10.1093/jleuko/qiae039 38417030 PMC11212797

[24] Peterson A, Bennin D, Lasarev M, Chini J, Beebe DJ, Huttenlocher A. Neutrophil motility is regulated by both cell intrinsic and endothelial cell ARPC1B. J Cell Sci. 2024;137(3):jcs261774. doi: 10.1242/jcs.261774 38224139 PMC10911274

[25] Lachmann N, Ackermann M, Frenzel E, Liebhaber S, Brennig S, Happle C, et al. Large-scale hematopoietic differentiation of human induced pluripotent stem cells provides granulocytes or macrophages for cell replacement therapies. Stem Cell Reports. 2015;4(2):282–96. doi: 10.1016/j.stemcr.2015.01.005 25680479 PMC4325194

[26] Brok-Volchanskaya VS, Bennin DA, Suknuntha K, Klemm LC, Huttenlocher A, Slukvin I. Effective and rapid generation of functional neutrophils from induced pluripotent stem cells using ETV2-modified mRNA. Stem Cell Reports. 2019;13(6):1099–110. doi: 10.1016/j.stemcr.2019.10.007 31708474 PMC6915846

[27] Frenz S, Conca R, Hesse S, Linder MI, Klein C. Induced pluripotent stem cell-derived neutrophil granulocytes - flow cytometry analysis reveals distinct subpopulations. Blood. 2021;138(1):3126. doi: 10.1182/blood-2021-150495

[28] Harper TC, Oberlick EM, Smith TJ, Nunes DE, Bray M-A, Park S, et al. GATA1 deletion in human pluripotent stem cells increases differentiation yield and maturity of neutrophils. iScience. 2023;26(10):107804. doi: 10.1016/j.isci.2023.107804 37720099 PMC10500457

[29] Schoen TJ, Calise DG, Bok JW, Giese MA, Nwagwu CD, Zarnowski R, et al. Aspergillus fumigatus transcription factor ZfpA regulates hyphal development and alters susceptibility to antifungals and neutrophil killing during infection. PLoS Pathog. 2023;19(5):e1011152. doi: 10.1371/journal.ppat.1011152 37126504 PMC10174577

[30] Gazendam RP, van de Geer A, Roos D, van den Berg TK, Kuijpers TW. How neutrophils kill fungi. Immunol Rev. 2016;273(1):299–311. doi: 10.1111/imr.12454 27558342

[31] Kienle K, Lämmermann T. Neutrophil swarming: an essential process of the neutrophil tissue response. Immunol Rev. 2016;273(1):76–93. doi: 10.1111/imr.12458 27558329

[32] Hopke A, Scherer A, Kreuzburg S, Abers MS, Zerbe CS, Dinauer MC, et al. Neutrophil swarming delays the growth of clusters of pathogenic fungi. Nat Commun. 2020;11(1):2031. doi: 10.1038/s41467-020-15834-4 32341348 PMC7184738

[33] Taylor PR, Brown GD, Reid DM, Willment JA, Martinez-Pomares L, Gordon S, et al. The beta-glucan receptor, dectin-1, is predominantly expressed on the surface of cells of the monocyte/macrophage and neutrophil lineages. J Immunol. 2002;169(7):3876–82. doi: 10.4049/jimmunol.169.7.3876 12244185

[34] van Bruggen R, Drewniak A, Jansen M, van Houdt M, Roos D, Chapel H, et al. Complement receptor 3, not Dectin-1, is the major receptor on human neutrophils for beta-glucan-bearing particles. Mol Immunol. 2009;47(2–3):575–81. doi: 10.1016/j.molimm.2009.09.018 19811837

[35] Thornton BP, Vĕtvicka V, Pitman M, Goldman RC, Ross GD. Analysis of the sugar specificity and molecular location of the beta-glucan-binding lectin site of complement receptor type 3 (CD11b/CD18). J Immunol. 1996;156(3):1235–46. doi: 10.4049/jimmunol.156.3.1235 8558003

[36] Clark HL, Abbondante S, Minns MS, Greenberg EN, Sun Y, Pearlman E. Protein deiminase 4 and CR3 regulate aspergillus fumigatus and β-Glucan-induced neutrophil extracellular trap formation, but hyphal killing is dependent only on CR3. Front Immunol. 2018;9:1182. doi: 10.3389/fimmu.2018.01182 29896200 PMC5986955

[37] Arya P, Kumar N, Bhandari U, Thapliyal S, Sharma V. Hidden attributes of zymosan in the pathogenesis of inflammatory diseases: a tale of the fungal agent. Iran J Basic Med Sci. 2023;26(4):380–7. doi: 10.22038/IJBMS.2023.67365.14770 37009011 PMC10008400

[38] Datta R, Miskolci V, Gallego-López GM, Britt E, Gillette A, Kralovec A, et al. Single cell autofluorescence imaging reveals immediate metabolic shifts of neutrophils with activation across biological systems. bioRxiv. 2024 2024.07.26.605362. doi: 10.1101/2024.07.26.605362 39211087 PMC11360992

[39] Lämmermann T, Afonso PV, Angermann BR, Wang JM, Kastenmüller W, Parent CA, et al. Neutrophil swarms require LTB4 and integrins at sites of cell death in vivo. Nature. 2013;498(7454):371–5. doi: 10.1038/nature12175 23708969 PMC3879961

[40] Tager AM, Luster AD. BLT1 and BLT2: the leukotriene B(4) receptors. Prostaglandins Leukot Essent Fatty Acids. 2003;69(2–3):123–34. doi: 10.1016/s0952-3278(03)00073-5 12895595

[41] Yamahashi Y, Cavnar PJ, Hind LE, Berthier E, Bennin DA, Beebe D, et al. Integrin associated proteins differentially regulate neutrophil polarity and directed migration in 2D and 3D. Biomed Microdevices. 2015;17(5):100. doi: 10.1007/s10544-015-9998-x 26354879 PMC4678772

[42] Li X, Utomo A, Cullere X, Choi MM, Milner DA Jr, Venkatesh D, et al. The β-glucan receptor Dectin-1 activates the integrin Mac-1 in neutrophils via Vav protein signaling to promote Candida albicans clearance. Cell Host Microbe. 2011;10(6):603–15. doi: 10.1016/j.chom.2011.10.009 22177564 PMC3244687

[43] Das J, Sharma A, Jindal A, Aggarwal V, Rawat A. Leukocyte adhesion defect: Where do we stand circa 2019? Genes Dis. 2019;7(1):107–14. doi: 10.1016/j.gendis.2019.07.012 32181281 PMC7063431

[44] Haist M, Ries F, Gunzer M, Bednarczyk M, Siegel E, Kuske M, et al. Neutrophil-specific knockdown of β2 integrins impairs antifungal effector functions and aggravates the course of invasive pulmonal aspergillosis. Front Immunol. 2022;13:823121. doi: 10.3389/fimmu.2022.823121 35734179 PMC9207500

[45] Wolach B, Gavrieli R, Wolach O, Stauber T, Abuzaitoun O, Kuperman A, et al. Leucocyte adhesion deficiency-A multicentre national experience. Eur J Clin Invest. 2019;49(2):e13047. doi: 10.1111/eci.13047 30412664

[46] Leal SM Jr, Vareechon C, Cowden S, Cobb BA, Latgé J-P, Momany M, et al. Fungal antioxidant pathways promote survival against neutrophils during infection. J Clin Invest. 2012;122(7):2482–98. doi: 10.1172/JCI63239 22706306 PMC3534057

[47] Tucey TM, Verma J, Harrison PF, Snelgrove SL, Lo TL, Scherer AK, et al. Glucose homeostasis is important for immune cell viability during candida challenge and host survival of systemic fungal infection. Cell Metab. 2018;27(5):988-1006.e7. doi: 10.1016/j.cmet.2018.03.019 29719235 PMC6709535

[48] Hellwig D, Voigt J, Bouzani M, Löffler J, Albrecht-Eckardt D, Weber M, et al. Candida albicans induces metabolic reprogramming in human nk cells and responds to perforin with a zinc depletion response. Front Microbiol. 2016;7:750. doi: 10.3389/fmicb.2016.00750 27242763 PMC4872603

[49] Häder A, Schäuble S, Gehlen J, Thielemann N, Buerfent BC, Schüller V, et al. Pathogen-specific innate immune response patterns are distinctly affected by genetic diversity. Nat Commun. 2023;14(1):3239. doi: 10.1038/s41467-023-38994-5 37277347 PMC10241821

[50] Li D-D, Jawale CV, Zhou C, Lin L, Trevejo-Nunez GJ, Rahman SA, et al. Fungal sensing enhances neutrophil metabolic fitness by regulating antifungal Glut1 activity. Cell Host Microbe. 2022;30(4):530-544.e6. doi: 10.1016/j.chom.2022.02.017 35316647 PMC9026661

[51] Silva-Gomes R, Caldeira I, Fernandes R, Cunha C, Carvalho A. Metabolic regulation of the host-fungus interaction: from biological principles to therapeutic opportunities. J Leukoc Biol. 2024;116(3):469–86. doi: 10.1093/jleuko/qiae045 38498599

[52] Zarbock A, Ley K. Neutrophil adhesion and activation under flow. Microcirculation. 2009;16(1):31–42. doi: 10.1080/10739680802350104 19037827 PMC2851240

[53] Winston DJ, Ho WG, Gale RP. Therapeutic granulocyte transfusions for documented infections. A controlled trial in ninety-five infectious granulocytopenic episodes. Ann Intern Med. 1982;97(4):509–15. doi: 10.7326/0003-4819-97-4-509 6751183

[54] Vamvakas EC, Pineda AA. Meta-analysis of clinical studies of the efficacy of granulocyte transfusions in the treatment of bacterial sepsis. J Clin Apher. 1996;11(1):1–9. doi: 10.1002/(SICI)1098-1101(1996)11:1<1::AID-JCA1>3.0.CO;2-F 8722714

[55] Price TH, Boeckh M, Harrison RW, McCullough J, Ness PM, Strauss RG, et al. Efficacy of transfusion with granulocytes from G-CSF/dexamethasone-treated donors in neutropenic patients with infection. Blood. 2015;126(18):2153–61. doi: 10.1182/blood-2015-05-645986 26333778 PMC4626256

[56] Shimizu K, Keller NP. Genetic involvement of a cAMP-dependent protein kinase in a G protein signaling pathway regulating morphological and chemical transitions in Aspergillus nidulans. Genetics. 2001;157(2):591–600. doi: 10.1093/genetics/157.2.591 11156981 PMC1461531

[57] Majumder A, Suknuntha K, Bennin D, Klemm L, Brok-Volchanskaya VS, Huttenlocher A, et al. Generation of human neutrophils from induced pluripotent stem cells in chemically defined conditions using ETV2 modified mRNA. STAR Protoc. 2020;1(2):100075. doi: 10.1016/j.xpro.2020.100075 33043305 PMC7543976

[58] Knox BP, Deng Q, Rood M, Eickhoff JC, Keller NP, Huttenlocher A. Distinct innate immune phagocyte responses to Aspergillus fumigatus conidia and hyphae in zebrafish larvae. Eukaryot Cell. 2014;13(10):1266–77. doi: 10.1128/EC.00080-14 24879123 PMC4187654

[59] Pachitariu M, Stringer C. Cellpose 2.0: how to train your own model. Nat Methods. 2022;19(12):1634–41. doi: 10.1038/s41592-022-01663-4 36344832 PMC9718665

[60] Sofroniew N, Lambert T, Evans K, Nunez-Iglesias J, Bokota G, Winston P, et al. Napari: a multi-dimensional image viewer for Python. Zenodo; 2022.

[61] Guzman EC, Rehani PR, Skala MC. Cell-analysis tools: an open-source library for single-cell analysis of multi-dimensional microscopy images. Imaging, Manipulation, and Analysis of Biomolecules, Cells and Tissues XXI. 2023;12383:82–6.

[62] Clasquin MF, Melamud E, Rabinowitz JD. LC-MS data processing with MAVEN: a metabolomic analysis and visualization engine. Curr Protoc Bioinformatics. 2012;Chapter 14:Unit14.11. doi: 10.1002/0471250953.bi1411s37 22389014 PMC4055029

[63] Melamud E, Vastag L, Rabinowitz JD. Metabolomic analysis and visualization engine for LC-MS data. Anal Chem. 2010;82(23):9818–26. doi: 10.1021/ac1021166 21049934 PMC5748896

[64] Warrick JW, Timm A, Swick A, Yin J. Tools for Single-Cell Kinetic Analysis of Virus-Host Interactions. PLoS One. 2016;11(1):e0145081. doi: 10.1371/journal.pone.0145081 26752057 PMC4713429

