# Supplementary figures and images for: GATA1-deficient human pluripotent stem cells generate neutrophils with improved antifungal immunity that is mediated by the integrin CD18
Source: PLoS Pathog. 2025 Feb 3;21(2):e1012654. doi: 10.1371/journal.ppat.1012654 (PMC11825098; doi:10.1371/journal.ppat.1012654)

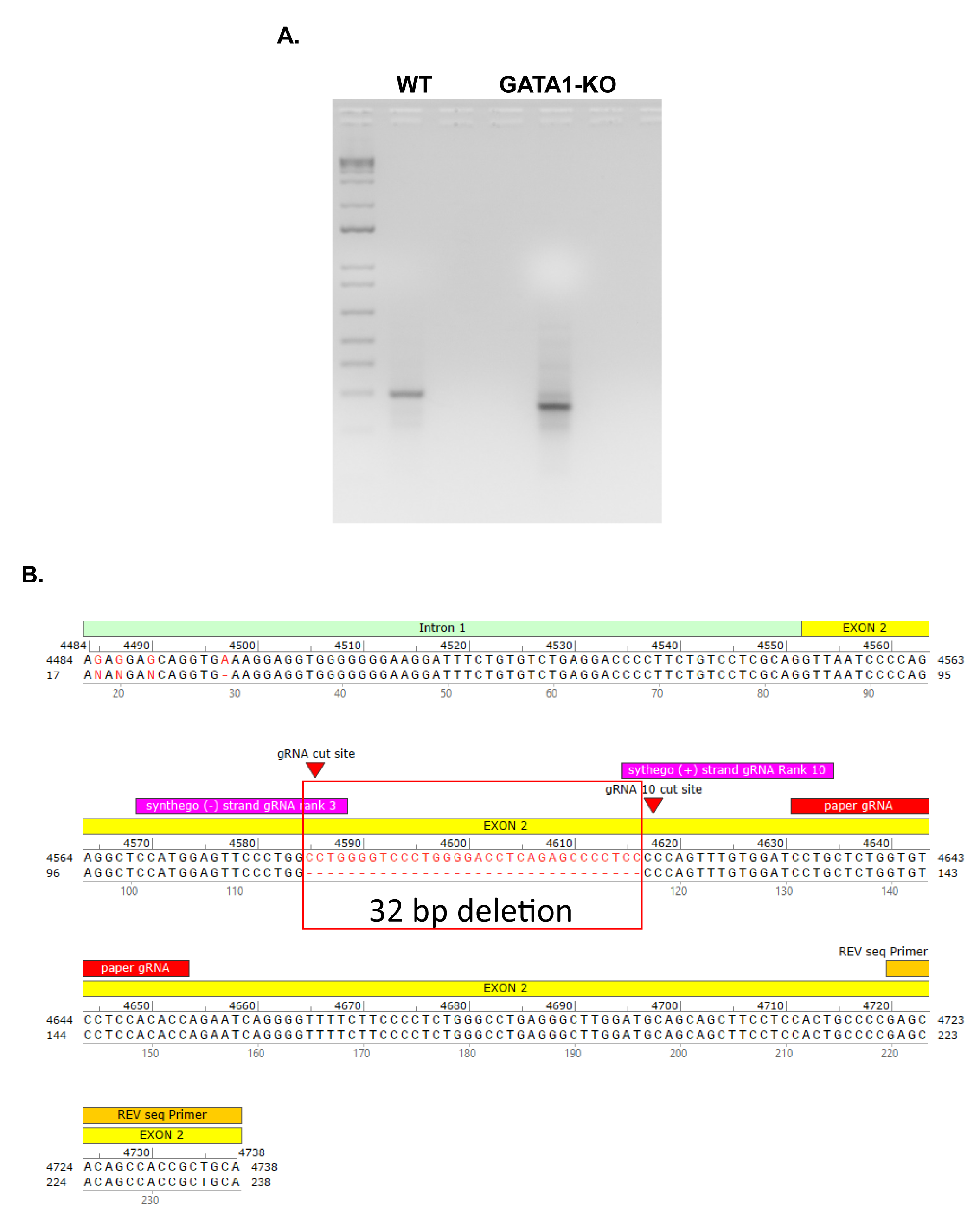

Supplement: S1 Fig — (A) Agarose gel showing a shift in the GATA1 gene following CRISPR-Cas9 mediated deletion of a 32 bp fragment in exon 2 of the coding sequence for the gene. (B) Sanger sequencing results confirming loss of the 32 bp fragment in exon 2 of the GATA1-KO mutant. (TIF) [file ppat.1012654.s001.tif]

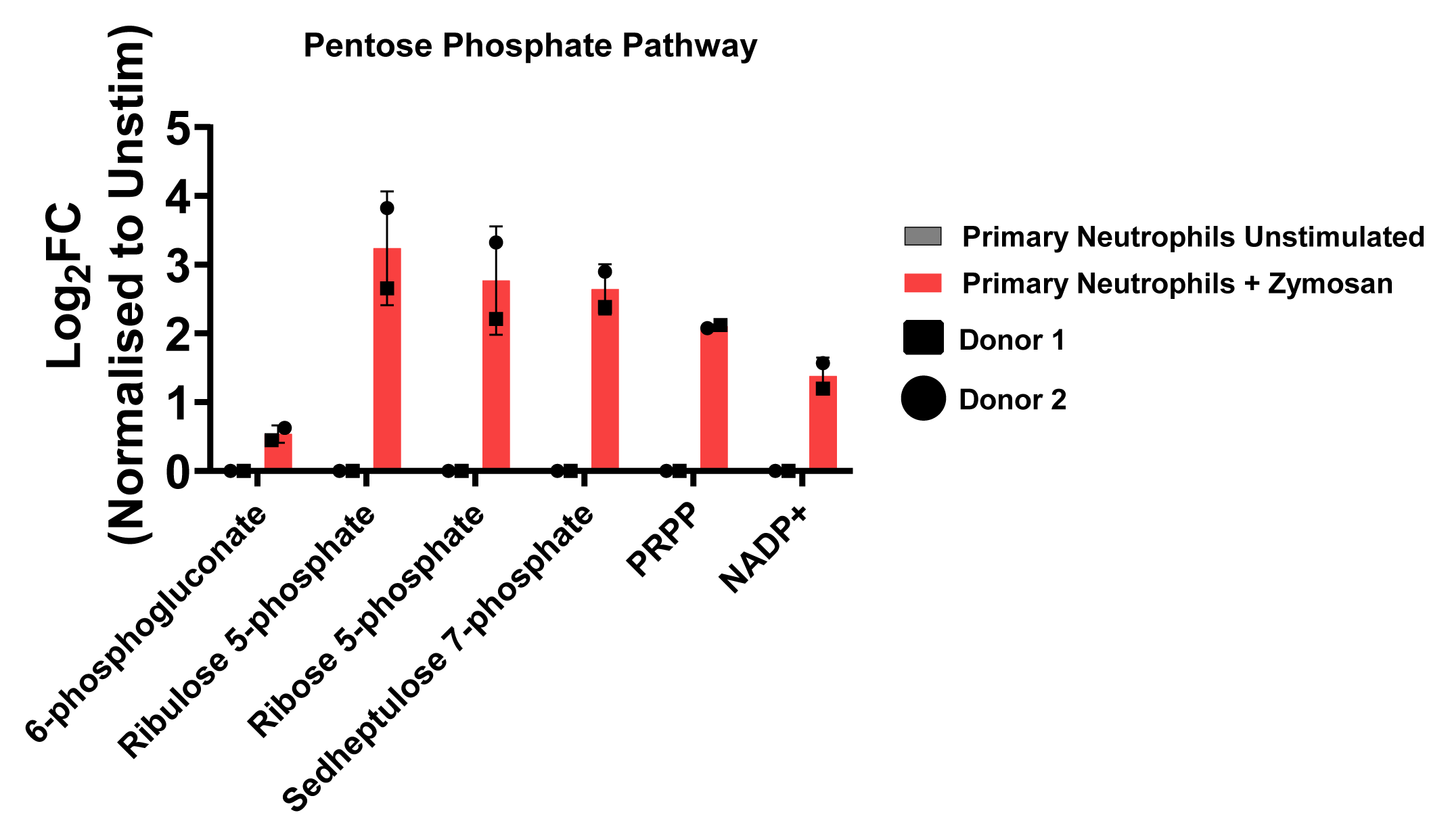

Supplement: S2 Fig — Fold-change in the abundances of metabolites associated with the pentose phosphate pathway. All samples are normalized to the unstimulated experimental group (n = 2 biological replicates from 2 independent donors). (TIF) [file ppat.1012654.s002.tif]

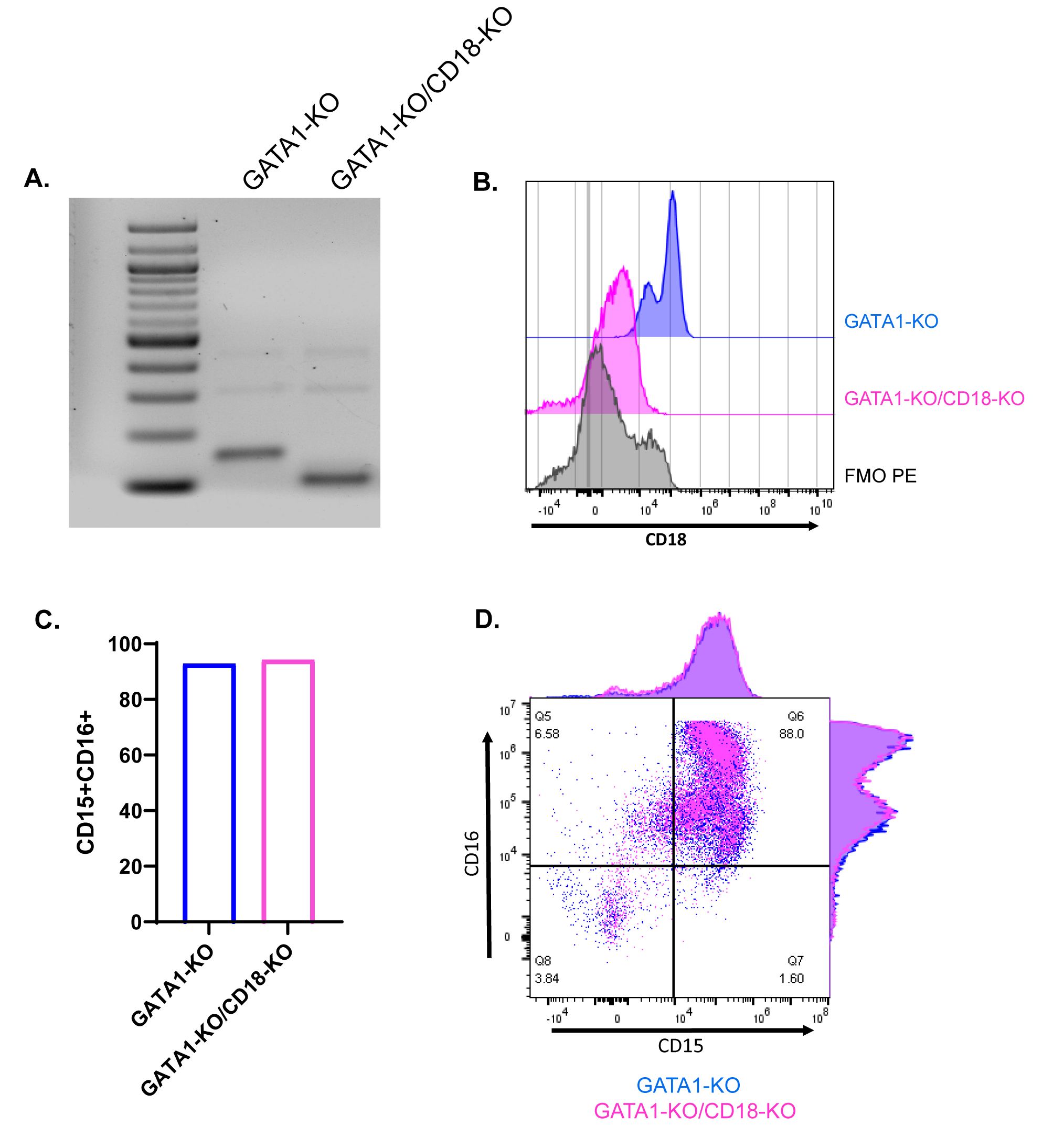

Supplement: S3 Fig — (A) Agarose gel showing a shift in the ITGB2 gene following CRISPR-Cas9 mediated deletion of a 52bp fragment in exon 4 of the coding sequence of the gene. (B) Representative histograms of surface marker staining for CD18 in GATA1-KO and GATA1-KO/CD18-KO iNeutrophils. Fluorescence minus one (FMO) PE is indicative of a negative control for CD18 staining where the anti-CD18-PE antibody was not added to the cells. (C) Quantification of the number of CD15+CD16+ cells within the live population of iNeutrophils. (D) Representative scatter plots of CD15 and CD16 expression intensities for GATA1-KO (blue) and GATA1-KO/CD18-KO (pink) iNeutrophils. (TIF) [file ppat.1012654.s003.tif]

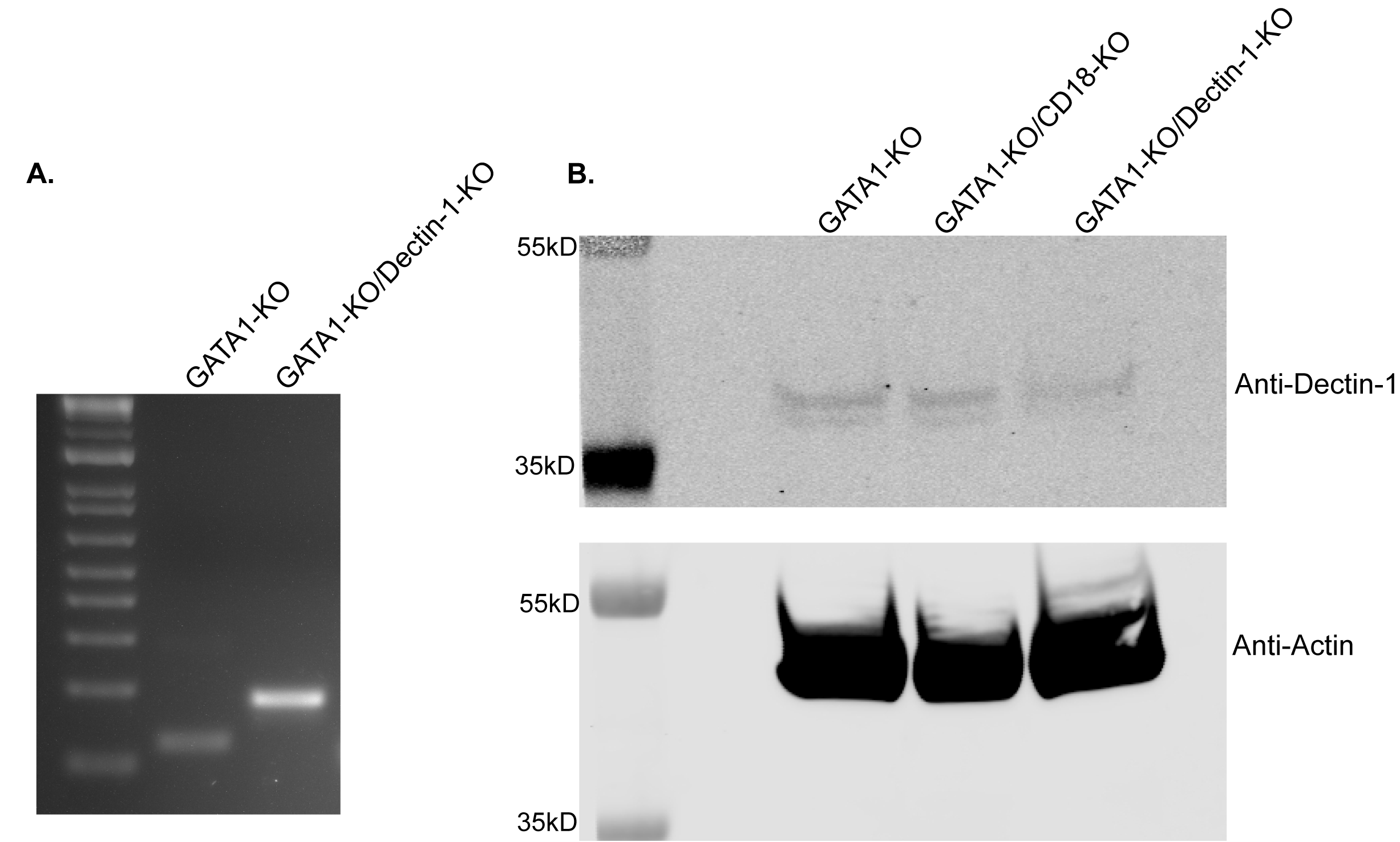

Supplement: S4 Fig — (A) Agarose gel showing a shift in the CLEC7a gene following CRISPR-Cas9 mediated deletion of a 63 bp fragment in exon 2 of the coding sequence of the gene. (B) Representative western blot showing dectin-1 protein levels in WT and KO iNeutrophils. (TIF) [file ppat.1012654.s004.tif]
