# Supplementary material for: GATA1-deficient human pluripotent stem cells generate neutrophils with improved antifungal immunity that is mediated by the integrin CD18
Source: PLoS Pathog. 2025 Feb 3;21(2):e1012654. doi: 10.1371/journal.ppat.1012654 (PMC11825098; doi:10.1371/journal.ppat.1012654)
Supplement: S1 Table — (DOCX) [file ppat.1012654.s008.docx]

**Table S1: Antibodies used in this study.**

| **Target** | **Fluor** | **Clone** | **Catalog #** | **Vendor** |
| --- | --- | --- | --- | --- |
| CD11b | PeCy7 | ICRF44 | 301321 | Biolegend |
| CD15 | APC-Fire 810 | W6D3 | 323058 | Biolegend |
| CD16 | BV711 | 3G8 | 563127 | BD Biosciences |
| BLT1R | BUV805 | 14F11 | 749044 | BD Biosciences |
| Clec7a (Dectin-1) | APC | 15E2 | 355405 | Biolegend |
| TLR2 | FITC | W15145C | 392307 | Biolegend |
| TLR4 | PE | HTA125 | 312805 | Biolegend |
| CD18 | PE | CBRLFA-1/2 | 366304 | Biolegend |
| CD32 | FITC | FUN-2 | 303204 | Biolegend |
| Zombie NIR | 746 | NA | 423105 | FischerScientific |
| Human TruStain FcX Fc Receptor Blocking Solution | NA | NA | 422302 | Biolegend |
| Ultracomp eBeads | NA | NA | 501129040 | ThermoFisher |
